# A Survey on U-Net with Variants and Their Applications in Two-Dimensional Medical Image Segmentations

**DOI:** 10.3390/jimaging12100497

**Published:** 2026-10-09

**Authors:** Tianhui Wu, Shamsher Ullah, Bowen Yu, Farhan Ullah, Jianghao Wang, Zhiqiang Gao, Kenji Yoshigoe, Yue Zhao

**Affiliations:** 1 Department of Computer Science, College of Science, Mathematics and Technology, Wenzhou-Kean University, Wenzhou 325060, China; 2 National Engineering Laboratory for Big Data System Computing Technology, School of Artificial Intelligence, Shenzhen University, Shenzhen 518060, China; 3 Cybersecurity Center, Prince Mohammad Bin Fahd University, Khobar 31952, Saudi Arabia; 4 Independent Researcher, Bangkok 10100, Thailand

**Keywords:** deep learning, U-Net, medical image segmentation, two-dimensional segmentation, medical imaging

## Abstract

Medical image segmentation plays a critical role in computer-assisted diagnosis, treatment planning, surgical navigation, and quantitative medical image analysis. Among deep learning-based segmentation architectures, U-Net has become one of the most influential frameworks because of its encoder–decoder structure and skip connections for integrating semantic and spatial information. This review focuses on U-Net and representative variants for two-dimensional medical image segmentation. We systematically analyze their architectural evolution, including attention-based designs, enhanced skip connections, deformable convolutions, and Transformer-based extensions and discuss their advantages, limitations, and application boundaries. Quantitative performance results are synthesized from published studies rather than reproduced through new benchmarking experiments; direct numerical comparisons are restricted to models evaluated within the same study or under clearly defined benchmark settings. We further relate architectural modifications to modality-specific challenges in MRI, CT, ultrasound, fundus imaging, dermoscopy, and microscopy. Finally, current challenges in computational efficiency, cross-domain generalization, data scarcity, interpretability, and clinical deployment are discussed, together with promising directions for future research.

## 1. Introduction

Medical image segmentation is a core task in medical image analysis that aims to delineate regions of interest, such as organs, tissues, and pathological structures, from medical images. Accurate segmentation supports a wide range of clinical applications, including disease assessment, treatment planning, and surgical guidance. However, medical images often exhibit low contrast, noise, and substantial anatomical variability, all of which increase the difficulty of precise segmentation. Traditional approaches, such as threshold-based segmentation, region growing, and edge detection, often lack sufficient robustness under these challenging conditions and may produce unreliable segmentation results. The development of deep neural networks has substantially changed this field. Ronneberger et al. proposed U-Net [1], an encoder–decoder architecture consisting of contracting and expanding paths connected by skip connections. This architecture effectively combines semantic information with fine-grained spatial features and has become a foundational framework for medical image segmentation.

The strong performance of U-Net in biomedical image segmentation benchmarks marked an important stage in the transition toward deep learning-based segmentation. Its influence extends beyond individual performance improvements because the encoder–decoder structure with skip connections established a highly extensible architectural paradigm for subsequent medical image segmentation research.

### 1.1. U-Net Improvements and Optimizations

Building on the original U-Net architecture, subsequent studies have mainly advanced along three directions: introducing attention mechanisms to improve target-focused feature selection, redesigning skip connections and feature-fusion pathways to reduce semantic mismatch, and integrating Transformer-based modules to strengthen long-range contextual modeling. These directions constitute the major architectural evolution of the U-Net family.

1. **Improvement based on attention mechanism**
  Attention U-Net [2]: In the original U-Net architecture, skip connections directly transfer encoder features to the decoder without explicitly determining their relevance to the target structure. When the segmentation target occupies only a small portion of the image, irrelevant background responses may therefore be propagated together with useful features, potentially reducing segmentation accuracy in complex anatomical backgrounds.
  To address this limitation, Attention U-Net [2] introduces attention gates that adaptively weight encoder features before they are transferred to the decoder. The gating mechanism emphasizes features associated with the target structure while suppressing irrelevant background responses. This mechanism is particularly useful when small organs or lesions are embedded in complex anatomical backgrounds because it improves target localization and reduces the propagation of irrelevant features through skip connections.
  However, attention gates introduce additional parameters and computational operations, which may increase inference cost and complicate deployment in resource-constrained or real-time clinical settings. Nevertheless, Attention U-Net represents an important extension of the original U-Net framework by introducing explicit feature-selection mechanisms.
2. **Deepening architecture and enhancing feature fusion**
  U-Net++ [3]: Although the skip connections of the original U-Net directly connect corresponding encoder and decoder stages, substantial semantic differences may remain between low-level encoder features and higher-level decoder representations. U-Net++ addresses this issue by introducing nested dense skip pathways with intermediate convolutional nodes. These pathways progressively transform and aggregate features across multiple scales, facilitating the integration of fine boundary information with higher-level semantic representations. However, the additional intermediate feature maps and nested pathways increase memory consumption and computational complexity, which may limit deployment on resource-constrained devices.
  Deformable U-Net [4]: A limitation of standard convolution is its fixed geometric sampling pattern, which may be insufficient for modeling irregular anatomical structures and highly variable target shapes. By introducing deformable convolutions with learnable spatial offsets, Deformable U-Net allows sampling locations and receptive fields to adapt to the geometry of the input features. This flexibility is particularly relevant to structures with substantial morphological variation and to thin or curved targets such as blood vessels.
  However, learning reliable deformation offsets introduces an additional optimization challenge. Poorly constrained offsets may overfit the training data or produce irregular sampling patterns, thereby reducing optimization stability and increasing training complexity.
3. **Integrating Transformer to Implement Global Context Modeling**
  TransUNet [5] combines convolutional feature extraction with Transformer-based global-context modeling. Convolutional neural networks are effective at capturing local spatial patterns but are inherently constrained by their local receptive fields. TransUNet addresses this limitation by incorporating a Transformer module that uses self-attention to model relationships among spatially distant image regions. Consequently, the network can combine local anatomical details with broader contextual information, which is particularly useful in multi-organ segmentation tasks where relationships among multiple anatomical structures are important.
  However, this design also introduces substantial computational and memory requirements because the cost of self-attention increases with the number of image tokens. This limitation becomes particularly important for high-resolution medical images. Therefore, balancing long-range contextual modeling with computational efficiency remains an important research challenge.
  Swin-Unet [6] adopts a different strategy for reducing the computational burden of Transformer-based segmentation. Instead of computing global self-attention over the entire feature map, it restricts self-attention to local windows and enables cross-window information exchange through the shifted-window mechanism. This design provides a practical balance between computational efficiency and long-range contextual modeling.
  This hierarchical window-based design improves the computational feasibility of Transformer-based segmentation while retaining multi-scale contextual modeling. However, the window configuration remains task-dependent and may require adjustment according to image resolution, target size, and dataset characteristics.
4. **Critical analysis of evolutionary trends**
  The evolution of U-Net and its variants reveals a persistent trade-off between segmentation capability and computational cost. Attention mechanisms, redesigned skip connections, deformable convolutions, and Transformer-based architectures address different limitations of the original U-Net, particularly in tasks involving small targets, complex boundaries, irregular geometry, or long-range contextual relationships. However, these architectural improvements are often accompanied by increased computational or memory requirements.
  As network architectures become deeper and more complex, their demands for GPU memory and computational resources increase, often reducing inference efficiency in practical applications. Moreover, no single U-Net variant has demonstrated consistently superior performance across all medical image segmentation tasks. The effectiveness of a particular architecture depends strongly on the imaging modality, anatomical target, dataset distribution, and experimental setting, and performance may therefore decrease when a model is transferred across domains or tasks.
  This task dependence reflects the substantial heterogeneity of medical imaging devices, anatomical structures, pathological appearances, and clinical requirements. Consequently, model development should consider both cross-domain generalization and task-specific architectural adaptation rather than assuming that a single architecture will be optimal for all scenarios. Specialized and computationally efficient designs may therefore be required for applications with distinct imaging characteristics or anatomical targets.

### 1.2. The Contributions of This Paper

Although previous studies have reviewed U-Net and its applications in medical image segmentation, there remains a need for a focused synthesis of its architectural evolution, quantitative evidence, and application boundaries in two-dimensional segmentation tasks. Accordingly, this review systematically analyzes the development of U-Net and representative variants, with particular emphasis on architectural mechanisms, literature-reported performance, modality-specific applications, and remaining challenges. The main contributions are as follows:

1. **System architecture analysis**
  We summarize and classify representative U-Net variants according to their architectural mechanisms and development trajectories. Beyond describing structural changes, this review analyzes the design motivations, technical mechanisms, and trade-offs associated with attention, redesigned skip connections, deformable convolutions, and Transformer-based extensions.
2. **Structured synthesis of literature-reported performance**
  We systematically synthesized quantitative performance results reported in representative studies across different imaging modalities and anatomical targets. Rather than directly ranking results obtained from heterogeneous datasets, quantitative comparisons were organized according to their experimental context. Direct comparisons were restricted to models evaluated on the same dataset under clearly defined settings, allowing architectural differences to be interpreted together with dataset and modality characteristics.
3. **Critical analysis of current challenges and future directions**
  We further critically examine major challenges in current U-Net research, including computational efficiency, model interpretability, data scarcity, cross-domain generalization, and clinical deployment. Based on these challenges, we discuss promising research directions involving lightweight architectures, multimodal information integration, data-efficient learning, explainable segmentation, and clinically oriented model development.

By systematically synthesizing the architectural innovations, reported performance, application characteristics, and practical limitations of U-Net variants, this review aims to provide a structured reference for future model design, evaluation, and clinical translation in two-dimensional medical image segmentation.

### 1.3. Review Scope and Approach

This review examines the architectural evolution of U-Net and its representative variants for two-dimensional medical image segmentation. The scope and approach are defined by the following considerations.

**Baseline framework:** The original U-Net [1] serves as the architectural baseline for this review because its symmetric encoder–decoder structure and skip connections established a foundational paradigm for medical image segmentation and have been widely adopted as a starting point for subsequent architectural modifications.

**Variant selection criteria:** The U-Net family has grown substantially since its introduction, and an exhaustive enumeration of all published variants is beyond the scope of a single review [7,8,9,10,11,12,13,14,15,16,17,18]. Instead of attempting to cover every modification, this review focuses on variants that introduced a *qualitatively distinct architectural mechanism* addressing an identifiable limitation of the original U-Net. Specifically, the following variants were selected because each represents a different category of architectural innovation:

1. **Redesigned skip connections (U-Net++ [3]).** The original skip connections directly concatenate encoder and decoder features at the same resolution, but substantial semantic differences may remain between shallow encoder features and deeper decoder representations. U-Net++ introduces nested dense skip pathways that progressively transform and aggregate features across multiple scales, thereby reducing the semantic gap between encoder and decoder.
2. **Attention-based feature selection (Attention U-Net [2]).** Standard skip connections transfer all encoder features to the decoder without distinguishing between task-relevant and irrelevant information. Attention U-Net addresses this by integrating attention gates that selectively weight encoder features before fusion, suppressing background responses and emphasizing target-related regions.
3. **Deformable spatial sampling (DUNet [4]).** Conventional convolution uses a fixed geometric sampling grid, which limits adaptability to structures with irregular, elongated, or branching morphology. DUNet replaces standard convolutions with deformable convolutions, allowing the sampling locations to be learned from the input features and enabling the receptive field to adapt to the target geometry.
4. **Transformer-based global-context modeling (TransUNet [5] and Swin-Unet [6]).** Convolutional operations are inherently local, and modeling long-range dependencies requires stacking many layers, which may be computationally inefficient. Transformer-based architectures use self-attention to directly model relationships among spatially distant image regions. TransUNet adopts a hybrid CNN–Transformer design, while Swin-Unet employs a hierarchical shifted-window strategy to balance global-context modeling with computational efficiency.
5. **Enhanced feature propagation through dense and residual connectivity (DenseU-Net [19] and ResUNet [20]).** As network depth increases, gradient propagation and feature reuse can become less effective. DenseU-Net introduces dense cross-layer connections to promote feature reuse and improve gradient flow, while ResUNet incorporates residual blocks to facilitate the optimization of deeper architectures.

These variants were selected because each introduced a mechanism that has influenced subsequent research and because sufficient published quantitative evidence was available to support a structured comparison. The classification is therefore based on the *primary architectural mechanism* rather than on chronological order, application domain, or reported performance.

**Application scope:** U-Net variants have been applied across a wide range of medical imaging tasks [21,22,23,24,25]. This review focuses on two-dimensional slice-wise segmentation across six imaging modalities—MRI, CT, ultrasound, fundus imaging, dermoscopy, and microscopy—because these modalities represent the predominant application domains of U-Net-based segmentation and exhibit qualitatively different imaging characteristics that influence architectural requirements. Three-dimensional volumetric segmentation, video-based analysis, and non-medical applications are outside the scope of this review.

**Evidence interpretation framework:** Quantitative performance results reported in the literature are synthesized and compared according to the level of experimental comparability. We adopt a three-level evidence hierarchy, as detailed in Section 4.1: within-study controlled comparisons provide the strongest basis for direct numerical comparison, shared-benchmark comparisons are interpreted as qualified comparisons requiring explicit consideration of implementation differences, and independent-study results are treated as contextual evidence rather than as a global model ranking. This framework ensures that performance differences are interpreted within their experimental context and prevents heterogeneous cross-study results from being treated as a universal ranking of U-Net variants.

### 1.4. Organizational Structure of the Article

This article systematically presents the evolution context and technical overview of U-Net in two-dimensional medical image segmentation. The subsequent sections are organized as follows:

1. **Section Two:** This section reviews the evolution of traditional methods and deep learning methods for two-dimensional medical image segmentation, providing a necessary foundation for readers to understand the historical background and innovative value of U-Net’s proposal.
2. **Section Three:** This section examines the theoretical foundations and architectural evolution of U-Net, including its symmetric encoder–decoder structure, skip connections, and the major mechanisms introduced by subsequent variants. It also summarizes the design principles underlying the evolution of the U-Net family.
3. **Section Four:** This presents a structured synthesis of commonly used evaluation metrics and literature-reported performance results. It distinguishes within-study comparisons conducted under defined experimental conditions from study-specific results reported across heterogeneous datasets and protocols.
4. **Section Five:** This section analyzes the application of U-Net variants across different imaging modalities, including MRI, CT, ultrasound, fundus imaging, dermoscopy, and microscopy, and relates modality-specific segmentation challenges to representative architectural adaptations.
5. **Section Six:** Based on the previous analysis, we conduct a critical discussion of the existing research, point out the limitations of the current research, and put forward constructive prospects for future research directions and clinical translation paths.

## 2. Key Technologies for Two-Dimensional Medical Image Segmentation

### 2.1. Traditional Segmentation Methods

Two-dimensional segmentation is a fundamental task in medical image analysis because it provides spatial delineation of organs, tissues, lesions, and other structures required for quantitative analysis and clinical interpretation. Before the widespread adoption of deep learning, medical image segmentation primarily relied on conventional image-processing and computer-vision techniques. These methods typically use manually designed intensity, texture, edge, or region-based features together with predefined rules or optimization models. Although they are often computationally efficient and straightforward to implement, their performance can be sensitive to noise, low contrast, parameter selection, and anatomical variability [26,27,28,29,30,31,32,33,34].

According to the existing literature, traditional medical image segmentation methods can mainly be classified into the following categories:

1. **Thresholding:** Threshold segmentation is one of the most fundamental and classic image segmentation methods. Its core idea is to divide image pixels into two major regions, the foreground and the background, by setting a gray-scale threshold. However, this method is highly dependent on the gray-scale distribution characteristics of the image and often performs poorly when dealing with medical images with low contrast or obvious noise interference. For instance, in MRI or CT images, the gray-scale differences among various soft tissues are usually very subtle, making it difficult for threshold segmentation to achieve accurate tissue boundary division. Despite these limitations, threshold-based segmentation remains useful as a preprocessing step in selected applications because of its simplicity and low computational cost. Its sensitivity to intensity variation and noise, however, has motivated the development of more robust segmentation approaches.
2. **Edge-based Segmentation:** Edge-based segmentation identifies object boundaries from local intensity gradients. Common operators include Sobel, Prewitt, and Canny detectors. Detected edges can subsequently be linked or refined to approximate organ or lesion contours. However, these methods are sensitive to image noise and may fail to produce continuous closed boundaries when anatomical interfaces are weak, blurred, or structurally complex.
3. **Clustering-based Segmentation [35]:** Clustering methods classify image pixels through unsupervised learning, such as K-means clustering and fuzzy C-means (FCM) clustering. These methods do not require a large amount of prior knowledge and can effectively handle images with significant grayscale differences. However, in medical images, due to the complexity of grayscale and texture features, clustering results often require subsequent manual intervention or prior information guidance.
4. **Graph-based Segmentation [36]:** Graph-cut methods formulate segmentation as an energy-minimization problem in which image pixels or regions are represented as graph nodes and relationships between them are represented by weighted edges. Foreground and background regions are separated by minimizing an appropriately defined graph-cut energy. Although graph-based methods can incorporate global optimization, their computational cost may become substantial for large-scale or high-resolution images.

The emergence of deep learning has substantially changed two-dimensional medical image segmentation by enabling task-specific feature representations to be learned directly from data. The following section reviews representative deep learning-based segmentation methods, with particular emphasis on U-Net and its variants, and discusses their advantages and limitations relative to traditional approaches.

Overall, traditional medical image segmentation methods offer advantages in computational efficiency and implementation simplicity. However, their strong dependence on manually designed features, parameter settings, and predefined rules limits their robustness to noise, low contrast, and complex anatomical structures. With advances in computing power and deep learning, end-to-end neural network-based segmentation methods have therefore become increasingly prominent and have substantially improved the automation and representational capability of medical image segmentation.

Nonetheless, traditional segmentation methods remain useful for relatively simple or well-constrained tasks. Thresholding, for example, can provide rapid separation of foreground and background when intensity distributions are sufficiently distinct, while region-growing methods remain applicable in semi-automatic segmentation systems when suitable initialization and parameter settings are available. However, evidence in the literature indicates that such methods are less robust when applied to complex and heterogeneous medical images.

### 2.2. Deep Learning Methods

Deep learning has produced substantial advances in medical image segmentation. Unlike traditional approaches that rely on manually designed features, deep neural networks can learn multi-level feature representations directly from training data through end-to-end optimization. This capability enables them to model complex variations in organs, lesions, and tissue structures and has made deep learning a major methodological framework for two-dimensional medical image segmentation [37,38,39,40,41,42,43,44,45,46].

Deep learning-based segmentation models use multilayer neural networks to learn hierarchical representations through backpropagation. Earlier layers generally capture low-level visual patterns such as edges and textures, whereas deeper layers encode increasingly abstract structural and semantic information. Encoder–decoder architectures can further combine high-level semantic representations with fine spatial details, enabling an end-to-end mapping from an input image to a segmentation mask.

End-to-end representation learning reduces dependence on handcrafted features and manual rule design. Representative architectures include fully convolutional networks (FCNs) [47] and encoder–decoder networks. U-Net [1] extends the encoder–decoder paradigm through symmetric multiscale processing and skip connections, providing a highly extensible architectural foundation for subsequent medical image segmentation models.

To aid readers in navigating the extensive body of research, the prevailing deep learning-based segmentation models found in contemporary studies can be systematically classified into the following primary categories.

1. **Fully Convolutional Network [47]:** Fully Convolutional Networks (FCNs) represent a foundational milestone in end-to-end pixel-level semantic segmentation. By replacing fully connected layers with convolutional operations, FCNs preserve spatial organization and enable dense predictions for inputs of varying sizes. Upsampling operations are used to recover the spatial resolution reduced during convolution and pooling. The work of Long et al. [47] therefore established an important transition from patch-based classification toward end-to-end dense prediction and provided a direct architectural foundation for later encoder–decoder segmentation networks, including U-Net.
  The main advantages of FCN include its fully convolutional design and its ability to process variable-sized input images through a single forward pass. However, early FCN architectures have limited capability for recovering fine spatial structures, which may result in coarse or blurred segmentation boundaries. Their feature-fusion mechanisms are also less effective than those of later encoder–decoder architectures in integrating information across multiple spatial scales.
  Despite these limitations, FCNs have been applied to multiple medical imaging tasks, including MRI brain tumor segmentation, CT lesion analysis, and organ delineation. More importantly, the FCN paradigm established the technical basis for subsequent encoder–decoder segmentation architectures such as U-Net.
2. **Encoder–decoder Structure [48]:** Encoder–decoder architectures combine semantic feature extraction with spatial reconstruction. The encoder progressively extracts higher-level representations through downsampling, whereas the decoder restores spatial resolution through upsampling to generate pixel-level segmentation outputs. Skip connections between corresponding encoder and decoder stages further integrate low-level localization information with high-level semantic context, improving boundary recovery in medical image segmentation tasks that require preservation of fine spatial details [49].
  Within this framework, U-Net [1] became a widely used baseline for biomedical image segmentation because of its symmetric encoder–decoder architecture and skip connections. Subsequent variants, including Attention U-Net [2] and DUNet [4], introduced specialized mechanisms for feature selection and geometric adaptation. More recently, architectures such as TransUNet [5] and Swin-Unet [6] have incorporated Transformer-based representations to strengthen long-range contextual modeling.
  The encoder–decoder structure provides a practical balance between semantic abstraction and spatial reconstruction, while its modular design facilitates the integration of additional architectural components. Nevertheless, computational cost, class imbalance, and domain-specific data variability remain important challenges. Encoder–decoder networks therefore continue to be widely used in applications such as brain MRI segmentation, abdominal CT organ delineation, retinal vessel extraction, and skin lesion analysis.
3. **Segmentation Network Based on Attention Mechanism [50]:** Models such as U-Net improve accuracy by means of skip connections, but they simply fuse all encoder features without distinguishing their correlations with the target. This indiscriminate fusion may introduce redundant or noisy information, limiting performance in complex medical images. To overcome this, researchers introduced the attention mechanism, which allows the network to selectively emphasize informative regions or features while suppressing irrelevant ones.
  The core idea of attention-based segmentation networks is to assign greater importance to task-relevant spatial regions or feature channels while suppressing less informative responses. In practice, attention modules can adaptively weight spatial locations, channels, or intermediate feature representations within the segmentation network.
  Representative examples include Attention U-Net, which introduces attention gates into skip connections to emphasize features relevant to the target structure. Attention mechanisms also form the basis of Transformer-based segmentation models such as TransUNet [5] and Swin-Unet [6], where self-attention is used to capture global context and long-range dependencies. Lightweight attention modules can additionally be integrated into efficient convolutional backbones to balance feature-selection capability with computational cost.
  Attention-based segmentation networks can improve boundary delineation by suppressing irrelevant background responses, enhance sensitivity to small or irregular target structures, and be integrated flexibly into existing encoder–decoder architectures.
  However, attention mechanisms introduce additional computation and memory requirements and may become less reliable when training data are limited or strongly imbalanced. Their effectiveness therefore depends on the quality and diversity of the training data as well as the design of the gating or attention mechanism. Despite these limitations, attention-based segmentation has been widely investigated in brain MRI, abdominal CT, retinal vessel, and skin lesion segmentation because of its ability to emphasize task-relevant features.
4. **Transformer-based segmentation network [51]:** Transformer-based segmentation has become an important research direction because self-attention can model long-range relationships beyond the local receptive fields of conventional convolutions [52,53,54,55,56,57,58,59,60]. TransUNet combines a CNN encoder with Transformer-based global-context modeling, whereas Swin-Unet [6] constructs hierarchical representations using shifted-window self-attention. More recent general-purpose segmentation foundation models, such as SAM and medically adapted variants such as MedSAM, further illustrate the potential of large-scale pretraining and transferable representations for medical image analysis.
  The principal advantage of Transformer-based segmentation is its ability to model global contextual relationships, which can be valuable for complex anatomical structures and spatially distributed targets. However, self-attention introduces substantial computational and memory requirements, particularly for high-resolution images, and effective training may depend on large datasets or pretrained representations. Consequently, many medical segmentation studies adopt hybrid CNN–Transformer architectures to combine convolutional locality with long-range contextual modeling.
5. **Lightweight and efficient network:** As medical image analysis increasingly moves toward clinically feasible real-time applications, computational efficiency has become an important consideration alongside segmentation performance. This demand has motivated the development of lightweight segmentation networks.
  The primary objective of lightweight segmentation is to reduce computational cost, memory consumption, and inference latency while preserving acceptable segmentation performance. Current strategies generally follow two directions: designing efficient backbones using architectures such as MobileNet, ShuffleNet, or EfficientNet together with operations such as depthwise separable or group convolution, and compressing trained models through techniques such as pruning, quantization, or knowledge distillation.
  The main advantages of lightweight segmentation networks are low inference latency, reduced memory consumption, and lower energy requirements, making them suitable for integration into diagnostic devices, smartphones, and other embedded systems. These characteristics expand the potential use of AI-assisted segmentation in resource-constrained settings, including primary care facilities and handheld ultrasound devices. However, lightweight design also introduces trade-offs between computational efficiency and segmentation capability.
  Lightweight models may have reduced representational capacity when segmenting very small, structurally complex, or highly heterogeneous targets. Aggressive compression may also reduce segmentation accuracy or introduce optimization instability, making the balance between efficiency and predictive capability an important design consideration.
  In practical applications, lightweight segmentation networks have been successfully used in scenarios such as retinal vascular detection in mobile ophthalmology and skin lesion classification on handheld devices. These applications demonstrate that lightweight segmentation technology is becoming an important bridge between cutting-edge AI research and clinical practice.

Table 1 summarizes the architectural advantages, mechanism-specific limitations, and representative application scenarios of major U-Net variants.

Table 2 summarizes the major technical capabilities and practical challenges associated with deep learning-based medical image segmentation.

Despite these advances, the practical deployment of deep learning-based medical image segmentation remains constrained by several factors. Large models may require substantial computational and memory resources, while supervised training depends heavily on high-quality expert annotations. In addition, differences among institutions, scanners, patient populations, and imaging protocols can introduce domain shifts that reduce cross-center generalization. Limited model interpretability further complicates clinical validation and may reduce clinician trust in automated segmentation results.

Recent research has therefore explored lightweight architectures, model pruning, quantization, domain adaptation, few-shot learning, and explainable artificial intelligence to improve deployment feasibility, data efficiency, and robustness. Future progress should extend beyond incremental improvements in benchmark accuracy and place greater emphasis on reliable cross-domain generalization, computational efficiency, interpretability, and integration with clinical workflows [68].

### 2.3. Comparison Between Traditional Methods and Deep Learning Methods

Traditional and deep learning-based segmentation methods differ substantially in feature construction, optimization strategy, data requirements, and applicable scenarios. Traditional methods generally depend on manually selected parameters, such as thresholds, seed points, or energy-function coefficients, and often require domain-specific prior knowledge. They can remain effective for images with relatively clear structures and limited noise but are less adaptable to heterogeneous modalities, complex lesion morphology, and large variations in image quality.

Compared with traditional approaches, deep learning-based segmentation represents a methodological shift from handcrafted feature engineering toward end-to-end representation learning. Neural networks can learn hierarchical features directly from image data and jointly optimize feature extraction and segmentation prediction. U-Net exemplifies this paradigm through its symmetric encoder–decoder architecture and skip connections, which combine high-level semantic information with fine spatial details to support pixel-level boundary recovery.

Despite these advances, several challenges limit the practical deployment of deep learning-based segmentation. Large models can impose substantial computational and memory requirements, while supervised training often depends on costly expert annotations. Performance may also degrade when models are transferred across scanners, institutions, populations, or acquisition protocols because of domain shift. In addition, limited interpretability can hinder model validation and clinician trust. These factors collectively complicate the translation of high-performing research models into routine clinical environments.

Recent research has therefore increasingly explored lightweight architectures, domain adaptation, semi-supervised learning, and explainable artificial intelligence to improve the balance among segmentation performance, computational efficiency, data requirements, and clinical practicality. Traditional segmentation approaches remain valuable in selected settings but are limited by their dependence on manually designed features and predefined rules. Deep learning-based methods, particularly U-Net and its variants, provide greater representational flexibility and have become central to automated medical image segmentation and related clinical decision-support applications.

Future progress is likely to depend increasingly on the integration of multimodal data fusion and explainable artificial intelligence.

### 2.4. Methodological Transition from Traditional to Deep Learning-Based Segmentation

The evolution from traditional image-processing methods to deep learning represents a fundamental change in how segmentation features are constructed and optimized. Traditional approaches rely primarily on handcrafted intensity, edge, texture, or region-based rules, whereas deep learning models learn hierarchical representations directly from annotated data. This transition reduces the dependence on manually designed features and enables segmentation systems to model substantially more complex anatomical and pathological variations.

A second methodological change is the transition from independent feature extraction and post-processing stages toward end-to-end representation learning. Encoder–decoder architectures integrate semantic abstraction and spatial reconstruction within a unified optimization framework. In medical image segmentation, this is particularly important because accurate predictions require both high-level recognition of anatomical structures and preservation of fine spatial information at boundaries.

However, the transition to data-driven segmentation also changes the source of methodological limitations. Performance increasingly depends on the availability and quality of annotated data, the similarity between training and deployment distributions, and the computational resources required for model optimization. Consequently, progress in segmentation should not be assessed solely through improvements in accuracy. Robustness to domain shift, annotation efficiency, and deployment feasibility have become integral dimensions of modern medical image segmentation research.

These observations establish the methodological background for the subsequent discussion of U-Net. Rather than treating individual architectural modifications as isolated improvements, the following section examines how the U-Net framework addresses the central tension between semantic representation and spatial localization and how later variants extend this principle through targeted architectural modifications.

## 3. Theoretical Foundations and Evolution of U-Net

Since the introduction of U-Net [1], its architecture has undergone extensive modification for two-dimensional medical image segmentation. The symmetric encoder–decoder structure and skip connections established a flexible framework for combining semantic representation with spatial localization. Subsequent variants have introduced targeted mechanisms to address specific architectural limitations, including feature selection, multi-scale fusion, geometric adaptability, and long-range contextual modeling.

This evolutionary process provides a useful framework for understanding why different U-Net variants exhibit different strengths across segmentation tasks. The following subsections first describe the theoretical formulation of the original U-Net and then examine how representative architectural modifications extend its core design principles.

### 3.1. U-Net Theoretical Formulation

The original U-Net introduced an innovative encoder–decoder structure, which effectively integrates multi-scale context information by introducing skip connections and significantly improves the accuracy of medical image segmentation. In recent years, the deep learning architecture based on U-Net has made significant progress in the field of two-dimensional medical image segmentation. Researchers continuously improve the model’s performance by introducing new network structures or mechanisms. For example, Deformable U-Net [4] adopts deformable convolution to dynamically adjust the shape of the convolution kernel, enhancing the segmentation ability of complex morphological structures such as retinal blood vessels. Attention U-Net [2] introduces the attention mechanism, enabling the model to autonomously focus on key areas and suppress irrelevant information, thereby enhancing segmentation accuracy, while U-Net++ [3] significantly improves the fusion effect among features at different levels by optimizing the skip connection structure.

In addition to the U-Net series, other deep learning architectures have also demonstrated strong potential. Swin-Unet [6] is based on the Transformer architecture and uses self-attention to capture global context information, achieving strong performance in multi-organ segmentation tasks. TransUNet [5] combines the strengths of CNN-based feature extraction and Transformer-based global-context modeling in a hybrid architecture. These methods not only improve segmentation performance but also open new directions for solving complex medical image analysis problems.

From the perspective of feature extraction, the encoder of U-Net progressively extracts hierarchical representations through successive convolution and downsampling operations. This process expands the receptive field and captures high-level semantic information, but the associated spatial downsampling can lead to the loss of fine-grained details that are important for boundary-sensitive segmentation tasks. To address this limitation, U-Net introduces skip connections that directly transmit multi-scale encoder feature maps to the corresponding decoder stages, enabling feature reuse and the integration of local spatial details with global semantic context. This design not only effectively retains fine-grained spatial information from the encoder, but also integrates local details with global context through cross-layer feature complementarity. This mechanism provides the architectural basis for subsequent improved models such as Attention U-Net and U-Net++, and reflects the core design principle of balancing representational efficiency with segmentation accuracy.

The following is the theoretical basis of U-Net in medical image segmentation:

1. **Structural mapping function:** U-Net maps an input image $X\in R^{H\times W\times C}$ to a pixel-level output probability map. Here, *H* and *W* denote the spatial dimensions of the input image, *C* denotes the number of input channels, *K* denotes the number of output categories, and $f_{\theta }$ represents the parameterized mapping function of the network:
  $$f_{\theta }:R^{H\times W\times C}\to R^{H\times W\times K}.$$
2. **Downsampling module:** The encoder gradually extracts higher-level semantic representations through successive convolution and pooling operations. Let $Y^{\left(l-1\right)}$ denote the input feature map of layer *l*, $W^{\left(l\right)}$ and $b^{\left(l\right)}$ denote the convolution kernel and bias term, respectively, σ(·) denote the nonlinear activation function, and Pool(·) denote the downsampling operation. A simplified formulation can be written as
  $$Y^{\left(l\right)}=\mathrm{Pool}\left(\sigma \left(W^{\left(l\right)}*Y^{\left(l-1\right)}+b^{\left(l\right)}\right)\right).$$
3. **Upsampling module:** The decoder gradually restores spatial resolution while integrating semantic information with encoder features through skip connections. Let Up(·) denote the upsampling operation, and let $E^{\left(l\right)}$ denote the encoder feature map transferred through the corresponding skip connection. The decoder feature at layer *l* can be expressed in simplified form as
  $$Y^{\left(l\right)}=\sigma \left(W^{\left(l\right)}*\left[\mathrm{Up}\left(Y^{\left(l-1\right)}\right)⊕E^{\left(l\right)}\right]+b^{\left(l\right)}\right),$$
  where ⊕ denotes feature-map concatenation.
4. **Dice loss:** The Dice coefficient measures the overlap between the predicted segmentation and the ground-truth mask. Let $p_{i}$ denote the predicted probability of pixel *i*, $g_{i}$ denote the corresponding ground-truth value, and ϵ denote a small constant used for numerical stability. The Dice loss is defined as
  $$L_{\mathrm{Dice}}=1-\frac{2\sum_{i}p_{i}g_{i}+\epsilon }{\sum_{i}p_{i}^{2}+\sum_{i}g_{i}^{2}+\epsilon }.$$

### 3.2. U-Net Architectures

**Figure 1:**
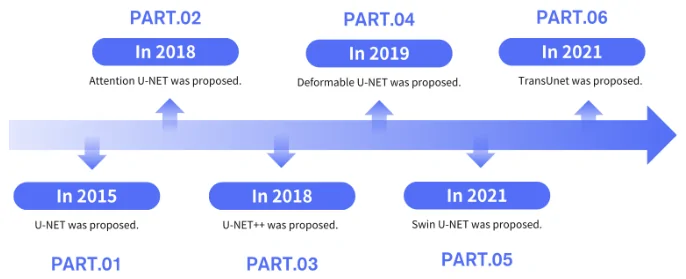
The Development History of U-Net and Its Variants.

Since U-Net was first proposed, its application and improvement in the field of two-dimensional medical image segmentation have gone through multiple important stages of development. These developments not only promote the advancement of medical image segmentation technology, but also lay a solid foundation for the application of deep learning in the medical field. This section will provide a detailed introduction to the evolution process of U-Net and its variants in chronological order and technological development stages. Figure 1 summarizes the chronological evolution of U-Net and its representative variants, while Table 3 provides an overview of major deep learning methods used in medical image segmentation.

The evolution process of U-Net can be roughly divided into the following stages, each accompanied by structural optimization and the introduction of new mechanisms, continuously promoting the improvement of segmentation accuracy.

1. **The Birth And Preliminary Applications of U-Net**
  U-Net was first proposed by Ronneberger et al. to address fine-grained biomedical image segmentation. Its principal architectural innovation is the symmetric encoder–decoder structure with skip connections that directly transfer spatially detailed encoder features to the corresponding decoder stages. This design reduces information loss during spatial reconstruction and improves the preservation of fine-grained structural details.
  U-Net demonstrated strong performance in the ISBI biomedical image segmentation challenge and showed that an encoder–decoder architecture with extensive data augmentation could achieve effective segmentation with limited annotated training data. Its success established U-Net as an influential baseline and provided an architectural foundation for numerous subsequent biomedical image segmentation methods.
2. **Exploration Of Initial Variants**
  In the early stage after the proposal of U-Net, researchers began to make targeted improvements to its architecture to deal with different segmentation challenges.
  Although the 3D U-Net [74] proposed by Cicek et al. is mainly aimed at three-dimensional volume data, its design concept also provides a new perspective for two-dimensional segmentation and expands the applicable boundaries of U-Net on data of different dimensions.
  Meanwhile, Oktay et al. introduced attention gates into the U-Net framework [2] and proposed Attention U-Net. Its principal modification is the integration of attention gates into skip connections, allowing encoder features to be selectively weighted before fusion with decoder features. This mechanism suppresses irrelevant background responses and emphasizes features associated with the target structure, thereby improving target localization in complex medical images.
  Overall, this stage of development reflects a transition from fixed feature transfer toward adaptive feature selection. The original Attention U-Net study demonstrated that attention gates can improve target localization by suppressing irrelevant feature responses, particularly in segmentation tasks involving small or spatially variable anatomical structures [2].
  With the continuous development of deep learning technology, variants of U-Net have gradually evolved towards more complex structures.
  Zhou et al. [3] demonstrated the effectiveness of nested dense skip connections through controlled comparisons with U-Net. For example, on the LiTS liver CT segmentation task, U-Net++ with deep supervision achieved an Intersection over Union (IoU) of 82.90%, compared with 76.62% for U-Net. Similar improvements were also observed for cell nuclei and colon polyp segmentation, although the magnitude of improvement varied across datasets.
  Jin et al. evaluated DUNet on several retinal vessel segmentation datasets, including DRIVE, STARE, CHASE_DB1, and HRF, demonstrating the applicability of deformable convolution to thin and geometrically irregular vascular structures [4].
3. **Introduction of Transformer Architecture (2021–present) [6,75,76]**
  With the development of deep learning, medical image segmentation methods have evolved from CNN based models to Transformer-based architectures. The earliest U-Net and its variants achieved significant success due to their effective capture of local features and skip connection structures. However, as task complexity increases, their limitations in handling long-range dependencies and global feature extraction have become apparent.
  Compared with purely convolutional architectures, Transformer-based models provide stronger explicit modeling of long-range dependencies through self-attention. This mechanism enables interactions among spatially distant image regions and can improve the representation of complex anatomical relationships. In addition, hierarchical Transformer architectures can combine information across multiple representation scales, providing complementary contextual information to local convolutional features.
  To combine convolutional locality with Transformer-based global-context modeling, several hybrid architectures have been developed. TransUNet [5] integrates Transformer representations with a CNN-based encoder–decoder framework, whereas Swin-Unet [6] employs hierarchical shifted-window self-attention to achieve multi-scale contextual modeling with reduced attention complexity. These developments illustrate an increasing trend toward complementary CNN–Transformer designs rather than the complete replacement of convolutional representations.

**Table 3:** Evolution of U-Net and its Variants in Medical Image Segmentation.

Evolutionary Stage	Core Driver	Representative Variants	Core Innovations	Achieved Effects	Persisting Challenges
Foundation	Effective utilization of limited annotated data	U-Net [1]	Symmetric encoder–decoder architecture; skip connection mechanism [49].	Solves segmentation with minimal annotated samples; achieves precise pixel-level localization.	Limited receptive field for long-range dependencies; semantic gap between encoder and decoder; fixed geometric structure limits deformation modeling.
Enhancement	Need for higher accuracy on details and boundaries	U-Net++ [3]; Attention U-Net [2]; DUNet [4]	Nested dense skip pathways [63]; attention gating mechanism; deformable convolutional [77].	Improves multi-scale feature fusion [70]; enhances small target segmentation [64]; improves segmentation of deformable organs.	Increased model complexity; attention mechanisms [50] unstable to train; sensitive to noise requiring careful tuning.
Transformation	Capturing long-range spatial dependencies	TransUNet [5]; Swin-Unet [6]	Hybrid CNN–Transformer architecture; pure Transformer with shifted windows [51].	Breaks CNN locality limitation [78]; achieves performance on multi-organ segmentation [79].	Extremely high computational and memory requirements; patchification loses spatial details; requires large training datasets.
Specialization	Meeting clinical deployment requirements [68]		Model compression and knowledge distillation; predictive uncertainty estimation; three-dimensional and temporal data modeling.	Improves computational efficiency; enhances result trustworthiness and interpretability; supports volumetric data analysis.	Trade-off between accuracy and efficiency; uncertainty quantification computationally expensive; three-dimensional models require more data and computing resources.

In summary, the characteristics of major deep learning models are systematically compared in Table 4. The theoretical foundation of U-Net is primarily built upon its symmetric encoder–decoder architecture combined with skip connections. The encoder–decoder design of U-Net strikes an effective balance between semantic understanding and detail retention. The encoder extracts high-level semantic features, while the decoder restores spatial accuracy through fine-grained information obtained from skip connections. This design enables the network to capture the global context without losing key structural details. As research has progressed, numerous innovations have been integrated into the U-Net framework, giving rise to many effective variants. These improvements address specific limitations: attention mechanisms adaptively focus on diagnostically relevant regions; dense skip connections promote multi-scale feature reuse; deformable convolutions enhance adaptability to irregular shapes; and Transformer-based modules effectively capture long-range dependencies.

From a methodological perspective, the evolution of U-Net reflects an iterative process in which new architectural components are introduced to address specific limitations while preserving the core encoder–decoder framework. Attention mechanisms improve feature selection, nested skip pathways reduce semantic mismatch, deformable convolutions increase geometric adaptability, and Transformer-based representations strengthen long-range contextual modeling. This problem-driven evolution explains the continued extensibility of the U-Net architecture.

Overall, the development of U-Net demonstrates the importance of a stable yet extensible architectural backbone. Its symmetric encoder–decoder structure and skip connections provide a modular framework in which specialized mechanisms can be incorporated without redesigning the entire segmentation pipeline. Consequently, later variants have been able to address task-specific limitations while preserving the fundamental principles of semantic abstraction and spatial reconstruction. This extensibility is one of the principal reasons why U-Net has remained influential across diverse medical image segmentation tasks.

### 3.3. Architectural Design Principles Derived from U-Net Evolution

The evolution of U-Net reveals several architectural principles that explain why the framework has remained influential in medical image segmentation.

1. **A stable encoder–decoder backbone enables extensible architectural design.** The original U-Net established a clear separation between semantic feature extraction in the encoder and spatial reconstruction in the decoder, while skip connections provide a direct pathway for recovering fine-grained information. This modular organization allows individual components to be modified without completely redesigning the segmentation framework. Consequently, later variants can extend the original architecture while preserving its fundamental localization–semantic integration mechanism.
2. **Most successful variants address a specific architectural bottleneck rather than merely increasing network complexity.** U-Net++ modifies skip pathways to reduce the semantic gap between encoder and decoder features; Attention U-Net introduces gating mechanisms to suppress irrelevant feature responses; and deformable convolution adapts the receptive field to geometrically irregular structures. These developments indicate that the evolution of U-Net is primarily problem-driven: architectural components are introduced to address identifiable limitations in feature fusion, feature selection, or geometric modeling [91,92,93,94,95,96,97,98,99,100].
3. **Architectural evolution increasingly relies on complementary mechanisms rather than complete replacement.** The emergence of hybrid architectures such as TransUNet illustrates that convolutional and Transformer-based representations provide complementary capabilities. Convolutional operations retain strong locality and spatial inductive bias, whereas self-attention improves long-range contextual modeling. The evolution of U-Net therefore reflects a broader trend toward integrating complementary representation mechanisms within a common encoder–decoder framework rather than replacing one architectural paradigm with another.

These principles provide an architectural interpretation of U-Net evolution. The following section shifts the focus from architectural development itself to evidence-based performance comparison and task-specific applicability of representative variants.

## 4. Performance Comparison and Application Scenarios of Different Variants

This section presents a structured evaluation of representative U-Net variants across different medical imaging tasks. The analysis is organized around the comparative methodology introduced in the following section, which distinguishes within-study controlled comparisons, shared-benchmark evaluations, and independent-study contextual evidence. The core architectural mechanisms examined include redesigned skip connections, attention gating, deformable convolutions, Transformer-based global-context modeling, and enhanced connectivity through dense and residual pathways. These mechanisms were introduced to address specific limitations of the original U-Net—semantic mismatch in skip connections, undifferentiated feature propagation, fixed geometric sampling, local receptive-field constraints, and limited gradient propagation in deeper architectures—each of which is discussed in detail in Section 3.

The following subsections first introduce the comparative framework and evaluation metrics, then examine the reported performance of representative variants grouped by architectural mechanism, and finally derive modality-specific insights and practical guidance for model selection.

### 4.1. Comparative Methodology and Evaluation Metric System

To improve the rigor and interpretability of quantitative comparisons, the evidence synthesized in this review is organized into three levels according to the comparability of the underlying experimental settings:

1. **Within-study controlled comparisons:** Results reported within the same study under a common experimental framework provide the strongest basis for direct numerical comparison. When different models are evaluated using the same dataset partition, preprocessing procedure, evaluation metric, and training protocol, the observed performance differences can be attributed more reliably to architectural differences. Therefore, this type of evidence is given the highest priority in the quantitative analysis of this review.
2. **Shared benchmark comparisons:** Public benchmarks provide a common test dataset and therefore improve comparability across models. However, training data, preprocessing, data augmentation, implementation details, and optimization strategies may still differ among studies or submissions. Consequently, results obtained on the same benchmark are interpreted as qualified comparisons rather than as fully controlled experiments. Public repositories and task-specific benchmark datasets also provide important infrastructure for reproducible comparison [101,102], while evaluation should consider standardized segmentation metrics and loss-function design [103,104].
3. **Independent-study contextual evidence:** Results reported in independent studies may involve different datasets, data splits, preprocessing procedures, training strategies, or evaluation protocols. Such results are useful for characterizing task-specific performance and application boundaries, but they are not used to establish a global numerical ranking among U-Net variants.

**Figure 2:**
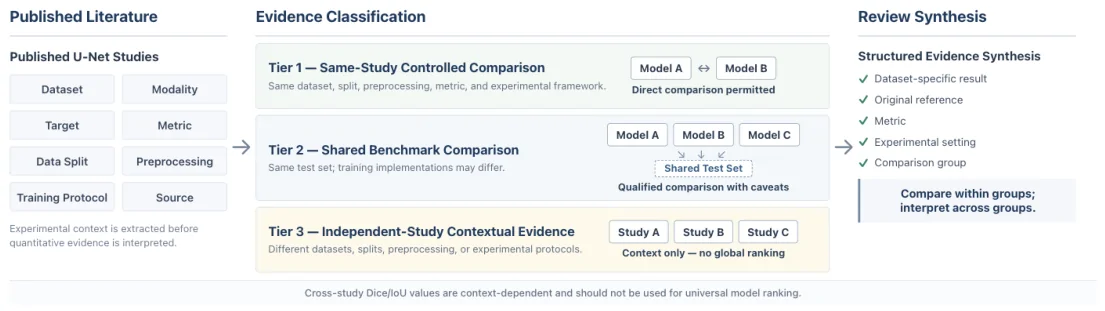
Evidence hierarchy and comparison framework used for synthesizing literature-reported performance of U-Net variants. Within-study controlled comparisons provide the strongest basis for direct numerical comparison, shared-benchmark results require explicit consideration of implementation differences, and independent-study results are interpreted as contextual evidence rather than as a global model ranking.

The evidence-selection and comparison strategy adopted in this review is summarized in Figure 2. This framework distinguishes controlled within-study comparisons from shared-benchmark evaluations and independent-study evidence, thereby preventing heterogeneous cross-study results from being interpreted as a universal ranking of U-Net variants.

Balancing between Evaluation Metrics and Clinical Decision-Making

1. **Precision and recall:** Precision and recall reflect different types of segmentation errors and should be interpreted according to the clinical objective. Increasing recall reduces false negatives and is therefore particularly important in screening tasks where missed lesions may have serious consequences. However, a strong emphasis on recall may also increase false positives and reduce precision. Conversely, applications that require accurate delineation of target structures, such as treatment planning or surgical navigation, place greater emphasis on precision and boundary accuracy. Therefore, the appropriate balance between precision and recall should be determined according to the clinical consequences of false-positive and false-negative predictions.
2. **Segmentation performance and computational efficiency:** Architectures with extensive attention or Transformer-based components can provide stronger contextual modeling but may also introduce substantial computational and memory requirements. In contrast, lightweight U-Net variants reduce parameter count, FLOPs, and inference latency at the potential cost of representational capacity. Model selection should therefore consider whether the target application prioritizes maximum segmentation performance under offline analysis or computational efficiency for real-time and resource-constrained deployment.

The performance discussions in this review are based on the multidimensional evaluation framework introduced above. A comprehensive model evaluation proceeds in several stages. First, the model must demonstrate sufficiently high segmentation accuracy, which is a fundamental requirement for practical application. Second, its stability and generalization ability under diverse data distributions should be examined to ensure reliable performance in real clinical environments. Third, the model’s computational efficiency must meet the requirements of the target deployment scenario, such as achieving adequate frame rates for real-time diagnostic tasks. Finally, the model should effectively address the most relevant practical challenges in the target application.

### 4.2. Core Architecture Innovation and Its Impact Analysis

The encoder–decoder backbone remains the structural foundation of the U-Net family. A major direction of subsequent development has been the optimization of feature transfer and fusion between encoder and decoder stages. Rather than replacing the overall architecture, many variants introduce targeted modifications to address limitations in semantic alignment, feature selection, geometric adaptability, or long-range contextual modeling.

1. **U-Net [1]**: The original U-Net combines high-level semantic representations with fine-grained spatial information through encoder–decoder skip connections. This design is effective for preserving localization information during spatial reconstruction. However, conventional convolutions provide limited explicit modeling of long-range dependencies, while direct same-scale skip connections may transfer semantically mismatched or redundant low-level features to the decoder.
2. **U-Net++ [3]**: U-Net++ introduces nested dense skip pathways that progressively reduce the semantic gap between encoder and decoder feature maps and strengthen multi-scale feature fusion. Controlled comparisons reported by Zhou et al. [3] demonstrate the benefit of this design. For example, on the LiTS liver segmentation task, U-Net++ with deep supervision achieved an IoU of 82.90%, compared with 76.62% for U-Net, although the magnitude of improvement varied across datasets. The nested feature-fusion strategy is particularly relevant to tasks that require integration of fine boundary information with higher-level semantic representations.
3. **DenseU-Net [19] and ResUNet [20,85,86]:** DenseU-Net and ResUNet enhance the classic U-Net architecture by introducing Dense Connections and Residual Connections respectively, optimizing the feature propagation efficiency from different paths. ResUNet embeds residual blocks into the encoder–decoder structure, effectively alleviating the vanishing gradient problem in deep networks, making it possible to train deeper networks and ensuring the stability of the training process. DenseU-Net uses dense cross-layer connections to promote feature reuse and facilitate information propagation between shallow and deep representations, which can be beneficial for segmentation tasks involving complex textures and fine-grained structural information. These two connection mechanisms provide important ideas for the optimization of U-Net, promoting the improvement of model performance from two directions: constructing a deeper network and enhancing feature reuse.
  It should be pointed out that the architectural ideas of DenseU-Net and ResUNet have been fully verified in two-dimensional image segmentation. Because the dense connection and residual connection mechanisms are naturally suitable for capturing the spatial context information of three-dimensional volume data, they have subsequently been widely expanded and applied in three-dimensional medical image segmentation. Dense connections improve gradient flow by enhancing feature reuse, while residual connections can alleviate the degradation problem of deep networks. Both exhibit significant advantages when dealing with stereoscopic images of complex anatomical structures. Since this article focuses on two-dimensional medical image segmentation methods, it will not further elaborate on DenseU-Net, ResUNet and their three-dimensional extended variants.

The attention mechanism achieves adaptive calibration of feature representations by assigning differentiated weights to different spatial positions and feature channels. This selective focus enables the network to concentrate on the regions with the richest information content while suppressing irrelevant or redundant parts, ultimately enhancing the feature recognition ability and segmentation performance of complex medical images.

1. **Attention U-Net [2]**: Attention U-Net [2] typically integrates attention gates in skip connections. These gates automatically suppress irrelevant background regions in the feature map passed from the encoder to the decoder. This is particularly effective in scenes with cluttered backgrounds or segmented small and complex structures.
2. **Application scenario:** In the task of cardiac MRI segmentation, Attention U-Net enables the model to precisely focus on the cardiac tissue area through its attention mechanism, while effectively ignoring the interference of irrelevant structures such as the surrounding thorax. This characteristic enables it to clearly delineate the anatomical boundary between the ventricle and the myocardium, significantly reducing the probability of tissue misdivision.

Attention gates can be integrated into existing encoder–decoder architectures to refine feature selection without changing the overall segmentation framework. They are particularly useful when target localization requires suppression of irrelevant background responses. However, the additional gating operations introduce extra computational and memory costs, and their effectiveness depends on the quality of the learned attention signals.

Deformable convolution significantly enhances the model’s ability to model irregular shapes by dynamically adjusting the receptive field of the convolution kernel [77]. This mechanism, by adaptively adjusting the convolution kernel, can more accurately fit complex anatomical forms, has advantages in segmenting variable structures, and has a relatively low computational overhead.

1. **Deformable U-Net [4]:** Deformable U-Net (such as DUNet) [4] replaces standard convolutional layers with deformable convolutional layers. This enables the convolutional kernel to dynamically adjust its sampling grid based on the input feature map, effectively learning geometric transformations and irregular shapes without additional supervision.
2. **Application scenario:** Deformable convolution is particularly relevant to structures with substantial geometric variability, such as retinal blood vessels. The original DUNet study evaluated the architecture on multiple public retinal vessel datasets, including DRIVE, STARE, CHASE_DB1, and HRF, and reported competitive global accuracy and AUC values, supporting the use of adaptive sampling for thin and irregular vascular structures [4].

Deformable convolution provides an adaptive mechanism for modeling irregular anatomical structures by allowing the sampling geometry to vary according to the input features. This property is particularly useful for thin, curved, or branching targets such as retinal vessels. However, deformable convolution primarily addresses geometric variability and does not by itself guarantee robustness across scanners, institutions, or acquisition conditions.

Transformer-based representations complement convolutional features by explicitly modeling long-range relationships through self-attention. This capability is particularly relevant to segmentation tasks in which the prediction of one anatomical structure depends on the spatial context of other structures, as in multi-organ segmentation.

1. **TransUNet [5]**: TransUNet combines convolutional feature extraction with Transformer-based global-context modeling. The CNN component preserves local spatial information, while the Transformer component models long-range relationships among image regions. This complementary design is particularly relevant to multi-organ segmentation tasks in which anatomical relationships extend beyond local receptive fields.
2. **Swin-Unet [6]**: Swin-Unet adopts a U-shaped Transformer architecture based on hierarchical shifted-window self-attention. On the Synapse multi-organ CT benchmark, Swin-Unet achieved an average DSC of 79.13% and an average Hausdorff Distance (HD) of 21.55. Under the same benchmark, U-Net, Attention U-Net, and TransUNet reported average DSC values of 76.85%, 77.77%, and 77.48%, respectively [6]. These results indicate competitive performance of hierarchical shifted-window modeling under this benchmark setting.

Transformer-based architectures are particularly relevant when segmentation requires long-range contextual information or relationships among multiple anatomical structures. However, their computational and memory requirements, data dependence, and implementation complexity can limit practical deployment. Future development should therefore emphasize more efficient attention mechanisms and complementary integration of local convolutional features with global contextual representations.

### 4.3. Comprehensive Analysis and Variant Selection Guide

Based on the above analysis, model selection should be guided by the imaging modality, target morphology, available training data, and computational constraints. Attention-based variants are particularly relevant when suppression of irrelevant background features is important, whereas deformable convolution is better suited to geometrically irregular or branching targets. U-Net++ provides an appropriate option when multi-scale feature fusion and reduction of the encoder–decoder semantic gap are priorities. Transformer-based variants can provide additional long-range contextual modeling when sufficient computational resources and suitable training or pretraining data are available. For resource-constrained applications, lightweight U-Net variants or efficient self-configuring baselines should be considered.

This systematic evaluation method helps to quickly narrow down the selection range in the early stage of research and focus the technical assessment on the most relevant performance dimensions. By structurally matching model characteristics with clinical needs in this way, researchers can ensure that the selected technical solutions not only perform well in terms of evaluation metrics but also meet the specific requirements of actual application scenarios. In conclusion, there is no absolute optimal solution for model selection. The core lies in precisely matching the advantages of the architecture with the actual challenges of the task.

1. **Is the target structure small or surrounded by complex backgrounds?** When the target structure is relatively small or embedded within complex surrounding tissues, conventional operations may fail to adequately highlight the region of interest, as the feature representations can be easily overwhelmed by irrelevant background information. In such cases, models augmented with attention mechanisms are preferable, since they enable the network to adaptively assign higher weights to salient regions while suppressing noise from non-essential areas, thereby improving the localization and segmentation of small structures in complex medical images.
2. **Does the target have a highly irregular or deformable shape, such as a vascular structure?** When target structures exhibit irregular, elongated, curved, or branching morphologies, fixed convolutional sampling patterns may be insufficient to represent their geometric variability. In such cases, deformable convolution can provide adaptive sampling locations that better follow the target geometry. This mechanism is particularly relevant to retinal vessels, airways, and other thin or morphologically variable structures. However, geometric adaptability should not be interpreted as automatic cross-domain generalization; robustness across patients, scanners, and acquisition protocols still depends on the diversity of the training data and appropriate domain adaptation or normalization strategies.
3. **Is it necessary to pay attention to the global context and multi-organ relationships?**
  When dealing with complex medical image segmentation tasks, accurately understanding the global context among multiple organs is particularly important. Due to their limited receptive fields, purely convolutional architectures often struggle to effectively capture long-range dependencies, which is a significant limitation for tasks requiring global contextual information. In contrast, the U-Net variant based on Transformer demonstrates unique advantages. Its core self-attention mechanism can directly establish the association of all regions within the entire image range, thereby better maintaining the consistency of the anatomical structure. Published studies indicate that global-context modeling can improve segmentation performance in tasks where long-range anatomical relationships are important.
4. **Choices in general scenarios and uncertain tasks**: When dealing with general segmentation tasks or when the application scenarios are not yet clear, we recommend giving priority to models with complex feature fusion mechanisms [70]. This type of model, with its strong feature integration capability, has demonstrated competitive results across a range of tasks.
  Among them, nnU-Net [105], as a widely recognized benchmark framework in the field of medical image segmentation, has a unique advantage in that it can automatically adapt to different types of datasets without requiring researchers to invest substantial effort in parameter tuning. In practice, mature self-configuring baseline frameworks such as nnU-Net can provide a stable starting point for task-specific model development while reducing manual configuration requirements.

The evolution of the U-Net architecture not only reflects the continuous progress at the technical level, but also marks the transformation of the research concept from merely pursuing benchmark performance to emphasizing systematic design with clinical practical value. Through a systematic analysis of the existing research results, we have reached a key conclusion: there is no “optimal model” applicable to all medical image segmentation tasks. The truly appropriate technology selection must comprehensively consider three key factors: the characteristics of the imaging mode, the features of the target anatomical structure, and the computing resource conditions in the actual application scenarios. This understanding will help promote the shift of this field from general model development to the design of precise solutions tailored to specific clinical needs.

Future development of U-Net-based medical image segmentation is likely to follow two complementary directions. The first is computational efficiency. Neural architecture search, knowledge distillation, pruning, quantization, and other model-compression strategies can help reduce inference latency and hardware requirements while preserving segmentation performance, which is important for applications such as bedside imaging and intraoperative guidance.

The second direction is deeper integration of contextual and multimodal information. Hybrid CNN–Transformer architectures, foundation models, and multimodal learning frameworks may enable segmentation systems to incorporate complementary information from imaging, radiomics, and other clinically relevant data sources. The objective is not merely to increase model complexity but to develop task-specific systems that balance segmentation accuracy, computational efficiency, robustness, and clinical usability.

Clinical translation requires segmentation models to satisfy requirements beyond benchmark accuracy. Robustness across institutions and acquisition conditions, computational efficiency, interpretability, and compatibility with existing clinical workflows are all important for practical deployment. Consequently, evaluation should consider not only segmentation metrics but also robustness, uncertainty, interpretability, latency, and clinical utility.

Successful deployment additionally requires collaboration among algorithm developers, clinicians, and healthcare information-system specialists. Such multidisciplinary evaluation can help determine whether model outputs are reliable, clinically interpretable, and compatible with safety, privacy, regulatory, and workflow requirements before large-scale clinical use.

Table 5 compares the major architectural features of representative U-Net variants.

### 4.4. Modality-Specific Application Insights

Through a systematic comparison of the performance and scenarios of each variant of U-Net, we have obtained the following core insights.

1. **Imaging modality strongly influences architectural requirements.** MRI segmentation is affected by non-standardized intensities and bias-field inhomogeneity, CT segmentation by low soft-tissue contrast and partial-volume effects, ultrasound by speckle and acoustic artifacts, and fundus imaging by thin branching structures and illumination variation. Architectural selection should therefore be informed by modality-specific image characteristics.
2. **Architectural modifications address segmentation-level consequences rather than acquisition artifacts themselves.** Attention mechanisms, multi-scale feature fusion, deformable convolution, and Transformer-based contextual modeling can improve feature representation, but they do not directly eliminate bias fields, CT noise, ultrasound speckle, or device-dependent domain shifts. Appropriate preprocessing and data harmonization remain necessary.
3. **The same architectural mechanism may have different practical value across modalities.** Deformable convolution is particularly relevant to geometrically variable structures such as retinal vessels, whereas Transformer-based global-context modeling is more relevant when relationships among multiple anatomical structures are important. Attention-based feature selection can be useful when target structures are surrounded by complex backgrounds.
4. **Clinical applicability depends on robustness beyond the development dataset.** Differences in scanners, institutions, patient populations, image quality, and acquisition protocols can alter model performance. Cross-center validation and modality-specific preprocessing should therefore accompany architectural evaluation before clinical deployment.

A major strength of the U-Net framework is its architectural extensibility. The encoder–decoder backbone allows attention mechanisms, deformable convolutions, Transformer-based modules, and other task-specific components to be incorporated without completely redesigning the segmentation pipeline. This modularity enables U-Net variants to be adapted to different imaging modalities, anatomical targets, and computational constraints, which has contributed to the continued use of the framework in medical image segmentation research.

**Figure 3:**
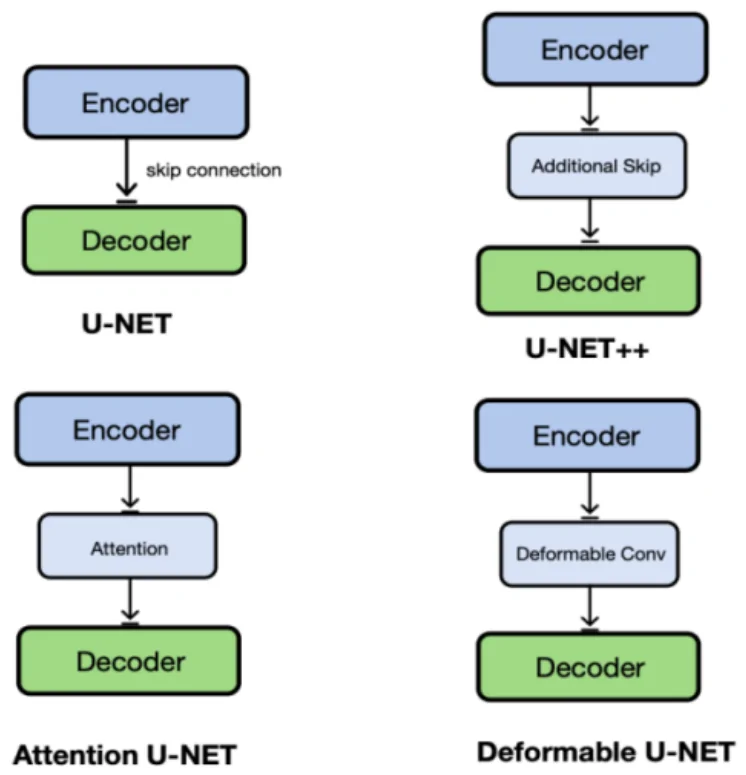
Schematic comparison of representative U-Net architectural modifications. Blue blocks denote encoder or intermediate feature-processing components, whereas green blocks denote decoder components.

Figure 3 schematically compares the major architectural modifications of representative U-Net variants.

## 5. Application of Two-Dimensional Medical Image Segmentation

Two-dimensional medical image segmentation supports a wide range of applications, including diagnosis, treatment planning, and surgical navigation [107,108]. Although several clinical tasks, such as liver or pulmonary nodule analysis, are commonly performed using three-dimensional CT volumes, this review focuses specifically on two-dimensional slice-wise segmentation, in which U-Net and its variants operate on individual image slices. The following subsections examine representative applications across different imaging modalities and relate their modality-specific challenges to corresponding architectural adaptations.

**Figure 4:**
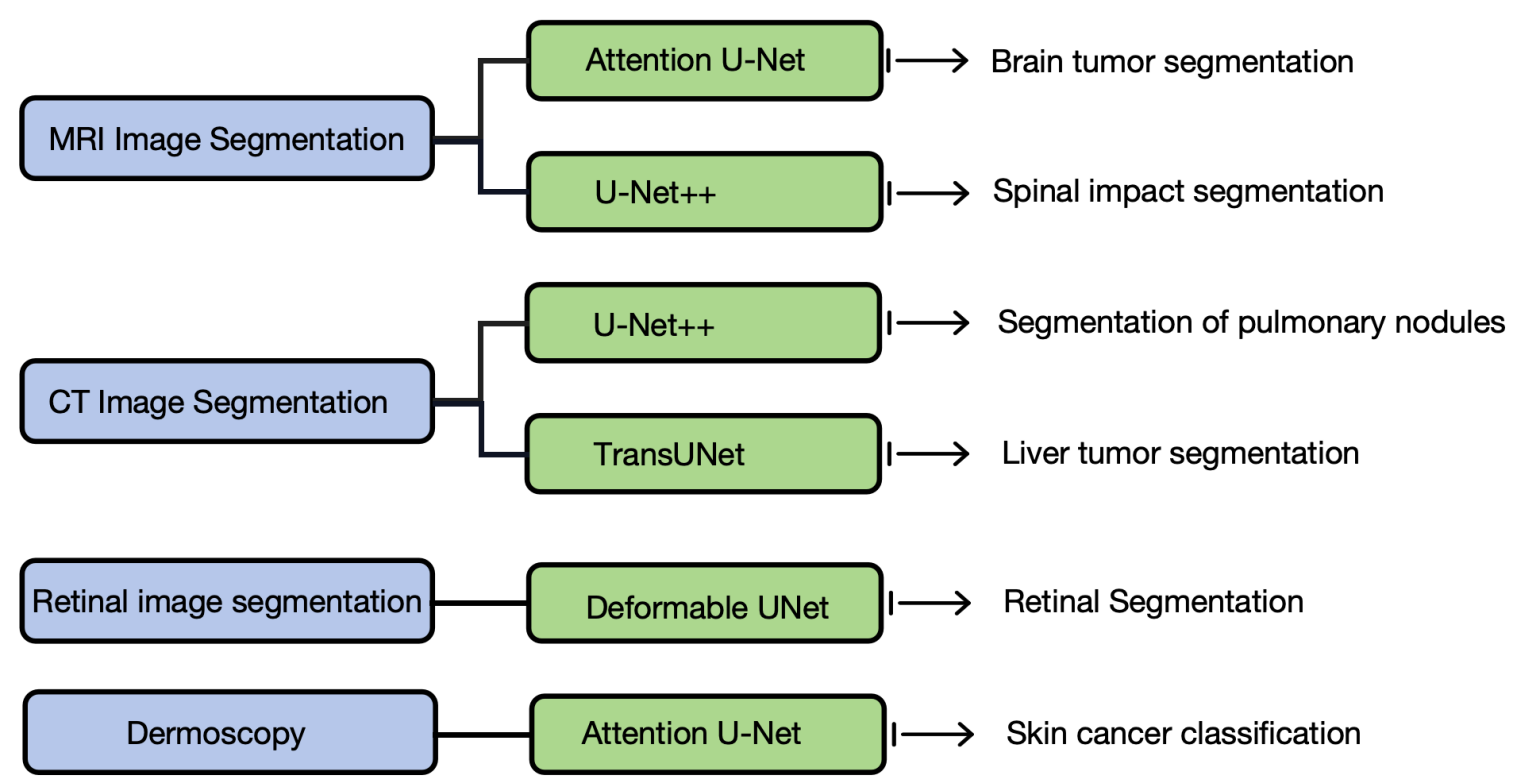
The application of deep learning in medical image segmentation. Blue blocks indicate imaging modalities, whereas green blocks indicate representative U-Net variants.

**Figure 5:**
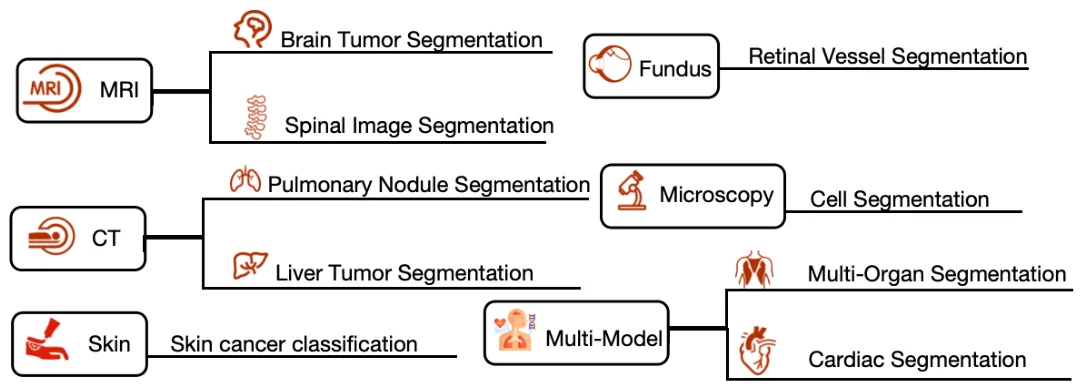
Deep learning applications in the medical image segmentation field.

Figure 4 and Figure 5 provide complementary overviews of representative segmentation architectures and their medical imaging applications. Together, they illustrate that architectural development is closely associated with the characteristics and requirements of specific imaging modalities and clinical tasks.

Medical imaging modalities differ substantially in their acquisition physics, noise characteristics, contrast mechanisms, and spatial appearance. Therefore, the suitability of a U-Net variant should not be determined solely by its overall segmentation accuracy. In this section, we analyze each modality according to a modality–challenge–architecture relationship: the dominant imaging characteristics are first identified, their consequences for segmentation are then examined, and finally the architectural modifications of representative U-Net variants are related to these specific challenges. Importantly, architectural modifications cannot completely compensate for acquisition-specific artifacts, and appropriate preprocessing, normalization, and data harmonization may still be required.

### 5.1. Magnetic Resonance Imaging Segmentation

Magnetic resonance imaging (MRI) [66,67] provides excellent soft-tissue contrast and is widely used in neurological, musculoskeletal, and other clinical applications [109,110,111,112,113,114,115]. Multi-sequence MRI provides complementary tissue information that can support more comprehensive characterization of anatomical structures and lesions. However, variability across sequences, scanners, acquisition protocols, and patients also creates substantial challenges for automated segmentation.

A modality-specific challenge of MRI is that image intensity does not have an absolute physical scale comparable to CT. In addition, magnetic-field and coil-sensitivity inhomogeneities can introduce slowly varying intensity nonuniformity, commonly referred to as the bias field. Such intensity variation may cause the same tissue type to exhibit different signal levels across spatial locations and can therefore interfere with automated tissue and lesion segmentation. Bias-field correction and intensity normalization are consequently important preprocessing steps for MRI analysis.

From an architectural perspective, attention gates are useful after such preprocessing because they suppress irrelevant feature responses and emphasize regions that are more informative for the target structure. This is particularly relevant for brain tumors whose boundaries may be weak or whose intensity overlaps with surrounding edema. Multi-scale feature fusion, such as that used in U-Net++, can further combine fine boundary information with higher-level semantic features. However, neither attention mechanisms nor dense skip connections directly correct the underlying MRI bias field; they should therefore be regarded as complementary to modality-specific preprocessing rather than as substitutes for intensity correction.

1. **Brain tumor segmentation**: Accurate brain tumor segmentation is crucial in medical image analysis, directly affecting diagnosis and treatment planning. Brain tumors typically present complex shapes and irregular boundaries in MRI images [68]. At present, it faces challenges such as low contrast between tumor tissue and healthy tissue, irregular morphology, and easy confusion with surrounding edema areas. To address these challenges, Attention U-Net [71] introduces attention mechanisms that enable the model to focus on the tumor region and effectively reduce interference from surrounding complex structures.
  Comparative analysis indicates that Attention U-Net [2] preserves tumor boundary details more effectively than FCN [47]. Compared with region-proposal-based methods, it does not require separate candidate region generation and screening and instead performs segmentation directly through attention-guided feature selection, improving both efficiency and accuracy. This improvement not only helps to more accurately determine the extent of the tumor, but also provides a more reliable basis for the development of subsequent treatment plans.
2. **Spinal image segmentation**: The diagnosis and treatment planning of spinal-related diseases require accurate segmentation of the fine structure of the spine. The presence of complex anatomical structures in spinal imaging increases the difficulty of segmentation [116].
  The challenges faced include complex spinal structures, significant individual differences, and low contrast between tissues such as surrounding nerves and blood vessels. Therefore, U-Net++ [3] provides a suitable architectural strategy for this type of task because its nested skip pathways progressively reduce the semantic gap between encoder and decoder features and aggregate information across multiple scales. This feature-fusion mechanism can be beneficial for spinal structures with substantial scale variation and weak boundaries. However, the effectiveness of the architecture remains dependent on the specific dataset and experimental protocol, and quantitative results reported in independent studies should not be directly compared.

### 5.2. Computed Tomography Image Segmentation

CT is widely used to diagnose tissues such as the lungs and liver due to its high resolution and 3D imaging capabilities [117,118,119,120,121,122,123,124]. In lung cancer screening, CT can detect small early nodules, but the diversity and complex background of lung tissue make accurate segmentation challenging for traditional methods [125].

**Figure 6:**
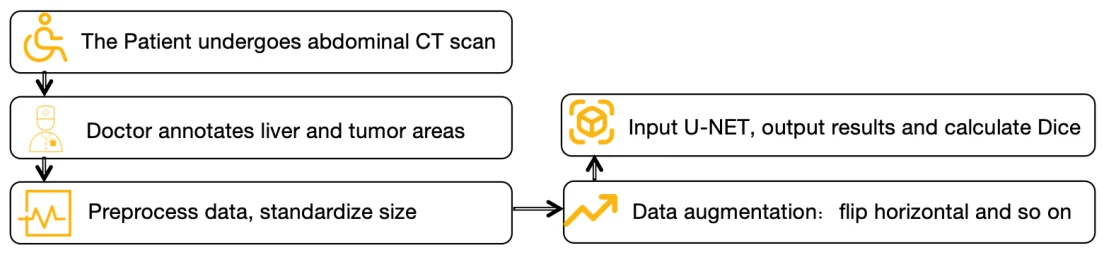
Workflow of CT-based liver tumor segmentation using U-Net. The process includes (1) CT scan acquisition, (2) manual annotation of liver and tumor regions, (3) data preprocessing and standardization, (4) data augmentation, and (5) U-Net inference with Dice evaluation.

Figure 6 illustrates the workflow of CT-based liver tumor segmentation using U-Net.

Compared with MRI, CT provides standardized intensity information through Hounsfield units, but segmentation is still affected by modality-specific problems such as low soft-tissue contrast, image noise, partial-volume effects, and acquisition or reconstruction variability. These factors are particularly problematic when lesions have attenuation values similar to the surrounding parenchyma, as commonly observed in liver-tumor segmentation.

Architectural modifications address different aspects of this problem. Dense and multi-scale skip connections in U-Net++ can preserve local boundary information and combine features at different receptive-field scales, which is useful for small lesions and poorly defined boundaries. Transformer-based variants such as TransUNet provide a complementary advantage by modeling long-range anatomical context, allowing the segmentation decision to incorporate relationships between a lesion and surrounding organs rather than relying only on local intensity differences. Nevertheless, these architectural mechanisms do not eliminate CT acquisition noise or intensity-windowing effects. HU windowing, normalization, denoising, and consistent preprocessing remain important when results are transferred across datasets or scanners.

1. **Pulmonary nodule segmentation**: The task faces challenges including large variations in nodule size, morphology, and density. To better integrate multi-scale features and enhance the recognition of small and blurry nodules, U-Net++ is employed for its dense skip connections. Furthermore, TransUNet [5] incorporates a Transformer architecture to excel at capturing complex textures. These architectures address complementary aspects of pulmonary nodule segmentation: U-Net++ emphasizes multi-scale feature fusion for local boundary recovery, whereas TransUNet introduces long-range contextual modeling through Transformer representations. Their reported quantitative performance should be interpreted within the corresponding experimental settings rather than compared across independent studies.
  Compared to traditional methods, U-Net++ [3] can effectively avoid misclassification and missed segmentation caused by uneven grayscale and diverse shapes. Compared with convolutional autoencoders, its encoder–decoder structure and skip connections can more effectively preserve image detail information.
2. **Liver tumor segmentation**: The segmentation of liver tumors plays a crucial role in the diagnosis and treatment of liver diseases.
  The challenge faced is that liver tumors have diverse morphological and density characteristics and may have low contrast with normal liver tissue. Therefore, TransUNet [5] combines the advantages of CNN and Transformer, achieving a balance between global semantics and local details through a hybrid architecture. This hybrid design is particularly relevant when local lesion appearance alone is insufficient for reliable segmentation and broader anatomical relationships are required for contextual interpretation.
  TransUNet [5] demonstrates superior performance over traditional segmentation approaches. It exhibits greater robustness to initialization and noise compared to level-set methods and eliminates the need for manual energy function definition required by graph cut algorithms through its automatic feature learning capability.

### 5.3. Ultrasound Image Segmentation

Ultrasound imaging differs fundamentally from CT and MRI because image formation is strongly affected by acoustic interactions between tissues. Its segmentation difficulty is commonly associated with speckle-like noise, acoustic artifacts, weak or discontinuous boundaries, low signal-to-noise ratio, and substantial variability across devices and operators [126]. Consequently, local intensity and edge information may be substantially less stable than in many CT or MRI segmentation tasks.

Attention-based U-Net variants provide one possible architectural response to these characteristics. Attention gates can reduce the propagation of irrelevant background responses through skip connections and emphasize feature regions that are more informative for the segmentation target. This may be beneficial when lesions are embedded in heterogeneous and noisy ultrasound backgrounds. However, attention mechanisms must be applied cautiously because weak target boundaries may also be suppressed if the learned gating signal is inaccurate.

Multi-scale architectures such as U-Net++ provide a complementary advantage by aggregating features across different receptive-field scales, which is useful when lesion boundaries are indistinct and target sizes vary considerably. This characteristic has motivated the application and comparison of U-Net and U-Net++ in breast ultrasound tumor segmentation [82,127,128].

Deformable convolution is another potentially useful mechanism when the target structure exhibits substantial geometric variation, because the learned sampling locations allow the receptive field to adapt to irregular boundaries. Nevertheless, deformable convolution should not be interpreted as a direct solution to ultrasound speckle noise. Noise suppression, appropriate augmentation, and device-aware normalization remain important because the acoustic artifacts originate from the imaging process rather than from the fixed geometry of conventional convolution alone.

### 5.4. Retinal Image Segmentation

Retinal imaging, as an important means of diagnosing ophthalmic diseases, provides a non-invasive window for observing the health of blood vessels and nerves. In this field, U-Net and its variants have become fundamental models and have performed particularly well in retinal vessel segmentation and pathological lesion detection. From the research perspective, these models, through precise analysis of vascular morphology, can effectively quantify disease progression and provide reliable evidence for the screening of diseases such as diabetic retinopathy and glaucoma. This not only promotes the development of computer-aided diagnostic technology, but also significantly enhances the efficiency and accuracy of clinical diagnosis.

The practical utility of retinal segmentation models also depends on their robustness to variations in image quality, illumination, acquisition devices, and vascular contrast. Improving such robustness is therefore important for the broader application of U-Net-based segmentation in computer-assisted ophthalmic analysis.

The challenge is that the blood vessels are slender, curved, and have complex branches, and the contrast with the background is low, making it difficult to capture the tips of small blood vessels. By introducing deformable convolutions, Deformable U-Net (DUNet) can dynamically adjust the position and shape of convolution kernels based on the geometric shape of blood vessels. DUNet [4] incorporates deformable convolution into the U-shaped architecture to adapt the receptive field to vessels with varying scales and geometries. Jin et al. evaluated DUNet on several public retinal vessel datasets, including DRIVE, STARE, CHASE_DB1, and HRF, demonstrating its ability to capture weak and geometrically irregular vascular structures. More recent controlled comparisons also support the benefit of deformable modeling: under a fixed split protocol on CHASE_DB1, DUNet achieved an F1-score of 80.37%, compared with 77.83% for the standard U-Net [129]. This modality–architecture relationship differs from that observed in MRI or ultrasound: deformable convolution primarily addresses the geometric variability of thin and branching vessels, whereas it does not directly correct intensity inhomogeneity or acquisition-specific noise.

**Figure 7:**
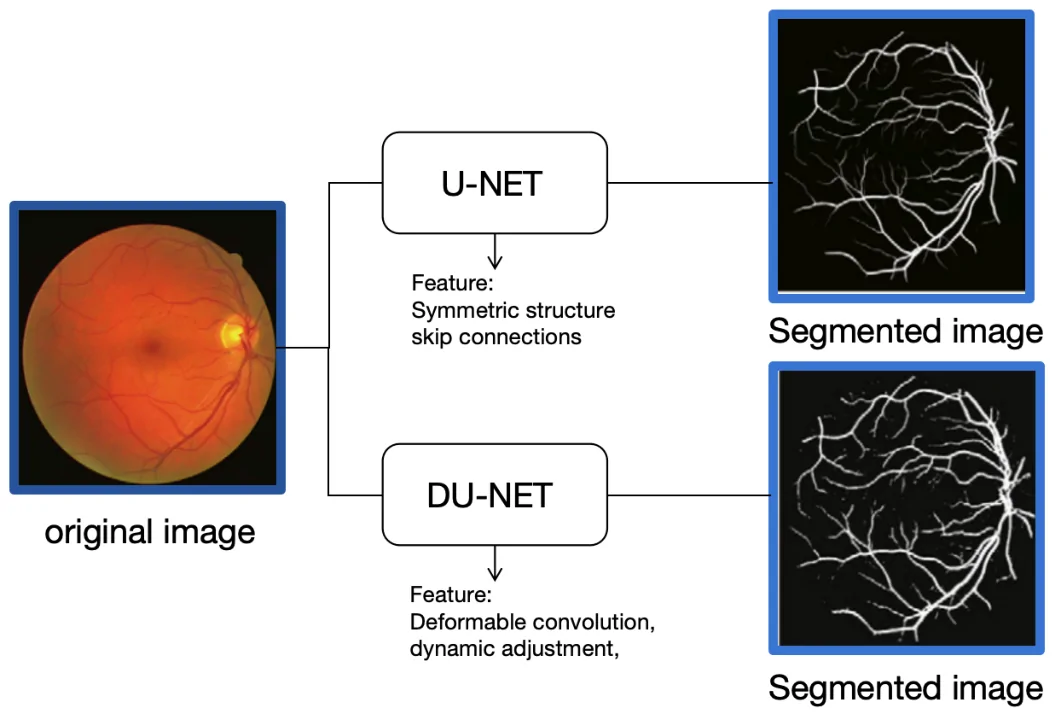
Comparison of segmentation results obtained with U-Net and DUNet for retinal vessel segmentation. Adapted from [4].

Figure 7 compares the segmentation results obtained with U-Net and DUNet for retinal vessel segmentation. Compared with traditional threshold segmentation and edge detection methods, DUNet can effectively avoid fragmentation and misclassification. Compared with the method based on multi-layer perceptron (MLP) combined with manual features, it can automatically learn features and adaptively adjust the feature extraction method through deformable convolution. Compared with a simple encoder–decoder network without skip connections, its skip connections can effectively preserve image details and avoid feature loss caused by downsampling.

### 5.5. Skin Image Segmentation and Classification

The application of U-Net and its variants in dermatoscopy [130,131,132], particularly on datasets like ISIC and HAM, focuses on a core clinical need, improving the efficiency and accuracy of skin cancer screening. By automating the tasks of lesion segmentation and classification, these models provide indispensable assistance in the initial review of images. This translates to a significant reduction in the workload for specialists, enabling them to prioritize high-risk cases and conduct screenings on a broader scale.

**Figure 8:**
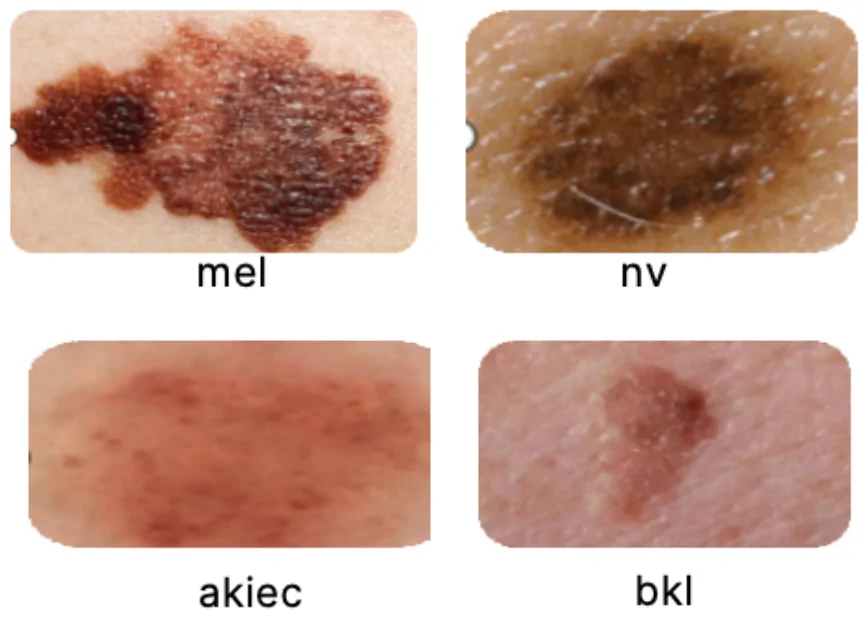
Representative misclassification examples from the HAM10000 dataset [133].

The challenge faced is the serious class imbalance problem in the HAM dataset, where different skin lesions (such as Mel and Nv, Akiec and BKL) have high similarity in color and edge texture, which can easily lead to misclassification. Introducing an attention mechanism into U-Net can reduce interference from complex backgrounds, while a joint learning strategy that uses U-Net for lesion-region extraction and CNN-based models such as ResNet or DenseNet for classification can improve the interpretability of the analysis. Although U-Net performs well in lesion segmentation, classification errors may still occur when lesion categories exhibit similar visual characteristics. Representative misclassification examples are shown in Figure 8. Melanoma (mel) may be misclassified as melanocytic nevus (nv) because of similarities in color and boundary texture, while actinic keratosis (akiec) may be confused with benign keratosis (bkl) when the available samples are limited or lesion morphology is ambiguous [73].

The segmentation capability of U-Net provides accurate regions of interest (ROI) for subsequent classification. Compared to directly classifying the entire image, this “segmentation classification” pipeline can effectively focus on the lesion itself, suppress irrelevant information, and has become the mainstream paradigm for dermatoscopy image analysis. Moreover, by enhancing interpretability and reducing false positives, this strategy facilitates more reliable clinical decision support and holds promise for integration into computer-aided diagnostic systems.

### 5.6. Microscopic Image Segmentation

Microscopic images are important tools in the research of pathology and cell biology, and U-Net and its variants have demonstrated outstanding performance in the segmentation of cell and tissue structures in this field.

This type of image usually faces challenges such as high cell density, blurred boundaries, noise interference and low contrast. DenseU-Net [19] deepens the network hierarchy by introducing a dense connection mechanism and promotes feature reuse, thereby capturing the fine boundary features of cells more accurately. Dense connectivity promotes extensive feature reuse and facilitates the propagation of fine-grained information across network layers, making DenseU-Net [19] particularly relevant to microscopic segmentation tasks involving dense structures and weak boundaries. Compared with the traditional gradient-based methods, this architecture can effectively overcome the segmentation deviation caused by blurred boundaries and complex internal textures.

Compared with the common fully connected network, its convolutional structure better retains the spatial information of the image and avoids the feature loss when expanding the two-dimensional image into a one-dimensional vector. These characteristics make DenseU-Net [19] particularly suitable for microscopic image analysis scenarios with high precision requirements, providing more reliable technical support for biomedical research. In summary, the U-Net architecture provides a solid foundation for microscopic image segmentation, and DenseU-Net [19] demonstrates significant advantages in cell segmentation tasks through its dense connection mechanism. This model can effectively overcome challenges such as blurred cell boundaries and high density, providing a more accurate and reliable quantitative analysis tool for biomedical research.

### 5.7. Emerging Application: Artificial Intelligence-Assisted Remote Medical Image Segmentation

With the rapid development of mobile healthcare and telemedicine, AI-assisted remote healthcare image segmentation has become an emerging application scenario with enormous potential and significant value. The challenges faced include limited remote device computing resources, network transmission delays, and the need for rapid feedback. Lightweight U-Net variants that incorporate efficient backbones such as MobileNet, ShuffleNet, or GhostNet are highly suitable for deployment in this scenario. These models are designed for mobile or remote medical devices with limited computational resources, enabling fast image segmentation and diagnostic feedback. This enables doctors to quickly and accurately analyze medical images captured in remote areas or home environments through the internet, enabling remote diagnosis and treatment recommendations and greatly promoting the rational allocation of medical resources and the convenience of medical services.

In summary, U-Net and its variants have demonstrated broad applicability across different two-dimensional medical image segmentation tasks. To provide a rigorous quantitative perspective, Table 6 summarizes representative performance results extracted from published studies. These values are not treated as a global ranking because segmentation performance depends strongly on the dataset, imaging modality, target structure, data partition, preprocessing procedure, and training protocol. Accordingly, comparisons are interpreted within their corresponding study-specific experimental settings.

Future progress in U-Net-based segmentation is likely to involve closer integration of multimodal information, more efficient model architectures, and improved interpretability. Multimodal learning can provide complementary information across imaging sources, while lightweight architectures can improve deployment feasibility on mobile or resource-constrained devices. Explainable and uncertainty-aware methods may further support clinical validation and user trust. Progress in these areas should be evaluated according to both algorithmic performance and practical clinical requirements.

Table 7 summarizes modality-specific imaging challenges and the corresponding architectural adaptations of representative U-Net variants.

### 5.8. Evidence-Based Principles for Model Selection

Through a comprehensive review of the application of U-Net and its variants in various medical imaging modalities, we have obtained the following core experiences and inspirations.

1. **The selection of a “problem-driven” model is the key to the success of clinical application:** The analysis in this section clearly indicates that no U-Net variant is omnipotent. The successful application stems from the precise analysis of the difficulties of specific clinical tasks: For brain tumors with ambiguous boundaries, the focusing ability of Attention U-Net is required; For retinal vessels with variable morphologies, the dynamic receptive field of Deformable U-Net is more effective; For abdominal CT segmentation that requires understanding the relationships among multiple organs, the global context modeling of TransUNet is of crucial importance. This means that model selection must be closely integrated with specific clinical problems. Therefore, the key to promoting the transformation of technology into clinical value lies in achieving an intelligent connection between advanced models and real diagnostic and therapeutic challenges.
2. **The modular design of U-Net is the basis of its clinical universality:** The core architecture of U-Net provides a modular backbone into which mechanisms such as attention gates, deformable convolutions, and Transformer-based representations can be flexibly incorporated. This design enables researchers to customize segmentation models according to the characteristics of different imaging modalities (such as MRI, CT, and fundus imaging) and anatomical structures, thereby facilitating the application of this technology across diverse clinical scenarios.
3. **There are still challenges from “laboratory precision” to “clinical availability”:** Although each variant achieved excellent Dice scores on specific datasets, they still face common challenges in real clinical settings: insufficient generalization to image data from different hospitals and devices; limited adaptability to rare cases or small-sample conditions; and limited transparency in the model’s decision-making process, which can affect doctors’ trust. Addressing these problems requires closer integration of algorithmic innovation and clinical knowledge.
4. **The future value lies in the deep integration with clinical workflows:** In the future, the value of medical image segmentation technology will increasingly be reflected in its deep integration with clinical workflows. This means that the research focus is shifting from merely pursuing segmentation accuracy to comprehensively enhancing the overall value of the technology in clinical practice. Specifically, this transformation is reflected in three key directions: developing lightweight models to support real-time application scenarios such as bedside diagnosis and telemedicine; exploring the fusion of multimodal data to provide a more comprehensive basis for clinical decision-making; and enhancing the interpretability of the model to transform it from a “black box” tool into a reliable decision-making assistant for doctors.

Overall, the success of U-Net and its variants proves that there is no universal and all-purpose model. What is truly effective are solutions specifically designed to address specific clinical challenges, such as network structures customized for specific problems like tiny lesions and complex boundaries. The key to future research lies in enabling these specialized models to further overcome practical challenges such as data heterogeneity and interpretability and truly integrate seamlessly into the clinical diagnostic process, thereby driving the technology from benchmark metrics toward tangible clinical value.

## 6. Summary and Future Research Directions

### 6.1. Summary

This paper systematically reviews the application progress of the U-Net structure and its main variants in two-dimensional medical image segmentation. It sorts out the theoretical basis and development background of U-Net and points out that its encoder–decoder structure with skip connection has become a classic design paradigm in this field. This infrastructure continues to drive the innovative development of medical image segmentation technology to this day.

Based on the structured synthesis of evidence reported in the literature, no single U-Net variant can be regarded as universally optimal across all segmentation scenarios. Different architectural modifications address distinct limitations of the original U-Net. U-Net++ [3,64] improves multi-scale feature fusion by redesigning skip pathways; attention mechanisms enhance the selection of task-relevant features; deformable convolutions improve adaptation to geometrically irregular structures; and Transformer-based architectures strengthen long-range contextual modeling. The practical value of each modification therefore depends on the imaging modality, target morphology, dataset characteristics, and experimental setting.

Across MRI, CT, ultrasound, fundus imaging, dermoscopy, and microscopy, the suitability of a U-Net variant depends strongly on modality-specific image characteristics and segmentation requirements. Table 8 summarizes the principal strengths, limitations, future directions, and representative application scenarios of major U-Net variants.

Despite substantial progress, important barriers remain, including limited annotated data, computational and memory requirements, domain shift across scanners and institutions, limited interpretability, and difficulties in clinical integration. Current strategies include lightweight model design, transfer and data-efficient learning, domain adaptation, multimodal integration, and explainable artificial intelligence. Addressing these challenges is essential for moving U-Net-based segmentation from controlled research settings toward reliable clinical deployment.

Although the U-Net series models have made remarkable progress, two-dimensional medical image segmentation still faces many challenges. As summarized in Table 8, these challenges also indicate several important directions for future research. Promising research directions include not only architectural innovation but also effective learning under limited labeled data, improved generalization across different devices and institutions, and computational efficiency suitable for practical clinical requirements. Addressing these issues will be essential for transforming segmentation models from research tools into reliable clinical support systems.

### 6.2. Future Research Directions

Based on the preceding analysis, several research directions are particularly important for advancing U-Net-based two-dimensional medical image segmentation.

1. **Lightweight and efficient architecture design:** Future research should further improve the balance between segmentation performance and computational efficiency. Promising strategies include neural architecture search, knowledge distillation, pruning, quantization, and hardware-aware network design. These approaches may facilitate deployment on resource-constrained clinical devices and support real-time applications such as bedside ultrasound and intraoperative image analysis.
2. **Enhancing cross-domain generalization:** Differences among institutions, scanners, acquisition protocols, and patient populations can substantially alter image distributions and reduce model performance after deployment. Future studies should therefore emphasize multi-center validation, domain adaptation, domain generalization, and harmonization strategies. Multimodal learning may provide additional complementary information when multiple imaging modalities are clinically available [140,141].
3. **Improving model interpretability and trustworthiness:** High segmentation accuracy alone is insufficient for clinical adoption. Future systems should provide interpretable evidence that allows clinicians to understand and verify segmentation outputs. Attention visualization, uncertainty estimation, counterfactual analysis, and clinically informed evaluation criteria may contribute to more transparent and trustworthy decision-support systems.
4. **Developing data-efficient learning methods:** Expert annotation remains expensive and time-consuming, particularly for rare diseases and small datasets. Few-shot learning, semi-supervised learning, self-supervised pretraining, active learning, and transfer learning provide potential strategies for reducing dependence on large fully annotated datasets [142,143]. These methods should also be evaluated under realistic distribution shifts to determine whether their benefits transfer across clinical environments.
  Recent developments in the broader medical image segmentation literature also include foundation-model adaptation, diffusion-based segmentation, Mamba-style sequence modeling, volumetric Transformer architectures, implicit neural representations, and scalable convolutional designs [144,145,146,147,148,149,150,151,152]. These directions are included here as emerging methodological context rather than as direct two-dimensional U-Net variants.
5. **Strengthening integration with clinical workflows:** Clinical translation requires more than algorithmic performance. Segmentation systems should be compatible with existing hospital information systems and imaging workflows, provide results in clinically interpretable formats, and satisfy requirements for reliability, latency, privacy, safety, and regulatory compliance. The role of such systems should be to support clinical decision-making rather than disrupt established diagnostic and treatment processes.

This review has traced the architectural evolution of U-Net and representative variants for two-dimensional medical image segmentation. The continued influence of U-Net arises from the extensibility of its encoder–decoder backbone, which has enabled subsequent methods to incorporate attention mechanisms, redesigned skip connections, deformable convolutions, residual and dense connectivity, and Transformer-based representations to address different segmentation challenges.

Future progress should extend beyond incremental improvements in segmentation accuracy. Greater emphasis is required on computational efficiency, data-efficient learning, robustness to domain shift, model interpretability, and integration with clinical workflows. These factors are essential for translating segmentation models from controlled research environments to reliable clinical applications.

Accordingly, future U-Net research should pursue task- and modality-specific architectural design while maintaining a balance among segmentation performance, efficiency, robustness, and clinical usability. Such a direction may facilitate the development of dependable medical image analysis systems that provide meaningful support for diagnosis, treatment planning, and other clinical decision-making tasks.

## Figures and Tables

**Table 1:** Architectural Advantages, Mechanism-Specific Limitations, and Application Scenarios of Representative U-Net Variants.

Method	Architectural Advantage	Mechanism-Specific Limitation	Representative Application Scenarios
U-Net [1]	The symmetric encoder–decoder architecture and skip connections preserve fine spatial information and enable effective training even with relatively limited annotated data.	Simple same-scale skip connections may transfer redundant or semantically mismatched low-level features to the decoder, while convolutional operations provide limited explicit modeling of long-range dependencies. These limitations become important when segmentation relies on global anatomical context or highly ambiguous boundaries [49].	Cell and nuclei segmentation; CT and MRI organ segmentation.
Deformable U-Net (DUNet) [4]	Deformable convolutions adapt the sampling locations and receptive fields to structures with irregular geometry, enabling improved modeling of thin, curved, or highly variable anatomical structures.	The additional offset fields introduce an extra optimization problem. Poorly learned offsets may generate irregular sampling patterns or overfit shape characteristics in limited or noisy training data, making optimization less stable and increasing training complexity.	Retinal vessel segmentation and other tasks involving elongated, branching, or geometrically variable structures.
Attention U-Net [2]	Attention gates selectively suppress irrelevant encoder features and emphasize task-relevant regions before skip-feature fusion, improving localization without requiring a separate localization network.	Attention gates depend on the quality of the learned gating signal; inaccurate or overly coarse attention may attenuate weak boundary features together with irrelevant background information. Moreover, gating mainly refines feature selection and does not by itself resolve the limited long-range dependency modeling of convolutional architectures.	Pancreas and abdominal organ segmentation; segmentation of small targets or targets surrounded by complex backgrounds.
U-Net++ [3]	Nested dense skip pathways progressively reduce the semantic gap between encoder and decoder features and facilitate multi-scale feature aggregation.	The nested pathways create multiple intermediate feature maps and segmentation branches, increasing activation memory and inference complexity. The benefit of deep supervision is also task-dependent, while aggressive pruning improves inference speed at the cost of segmentation accuracy.	Cell nuclei, colon polyp, liver, and other organ or lesion segmentation tasks.
Swin-Unet [6]	Hierarchical shifted-window self-attention provides multi-scale representations and improves the modeling of long-range contextual relationships compared with purely convolutional architectures.	Self-attention is computed within local windows, so information between distant regions is exchanged progressively across successive shifted-window blocks rather than directly. As a pure Transformer architecture, Swin-Unet also has weaker convolutional inductive bias and may require more data or training iterations, while high-resolution token processing still introduces considerable memory and computational demand [51].	Multi-organ CT segmentation and cardiac image segmentation.
TransUNet [5]	The hybrid CNN–Transformer architecture combines local feature extraction and precise localization from CNNs with global-context modeling from Transformer self-attention.	Patch tokenization and Transformer processing increase computational and memory requirements as the token sequence grows. Transformer features also contain less fine-grained localization information, making the architecture dependent on CNN skip features for boundary recovery; performance may additionally benefit substantially from large-scale pretraining [51].	Multi-organ CT segmentation and cardiac segmentation, particularly when relationships among multiple anatomical structures are important.
DenseU-Net [19]	Dense connectivity promotes feature reuse and provides short gradient paths between layers, improving feature propagation across different representation levels.	Repeated concatenation retains features from preceding layers, causing activation memory and data-transfer requirements to grow with network depth. This becomes especially expensive for high-resolution medical images and can increase both training time and inference latency.	Cellular and microscopic image segmentation and other tasks requiring extensive reuse of fine-grained features.
ResUNet [20]	Residual blocks improve gradient propagation and make deeper encoder–decoder architectures easier to optimize.	Residual connections primarily improve optimization rather than explicitly addressing multi-scale semantic mismatch, irregular geometry, or domain shift. Increasing residual depth also increases the parameter and activation burden and may increase overfitting risk when annotated medical data are limited.	Liver, brain tumor, skin lesion, and retinal structure segmentation.

*Note:* The limitations listed here refer to architecture-specific mechanisms and their practical consequences rather than generic disadvantages. Their impact may vary with dataset size, imaging modality, target morphology, preprocessing strategy, and implementation details.

**Table 2:** Advantages and Limitations of Deep Learning Methods in Medical Image Segmentation.

Category	Key Feature	Detailed Description	Advantages	Limitations
Technical Capability	Strong automatic feature learning [1,61]	Models learn feature representations directly from raw data.	Avoids handcrafted features, effective for complex structures and low-contrast images, improving accuracy.	Requires large annotated datasets for training, which are costly and time consuming.
Technical Capability	End-to-end automated segmentation [1]	Fully automated pipeline from images to masks.	Reduces manual intervention, shortens development cycles, integrates into clinical workflows.	High computational demand for training, challenging on resource-constrained devices.
Technical Capability	Skip connections [1,49,62,63]	Links encoder and decoder layers at the same resolution to preserve spatial information.	Improves localization accuracy and helps capture small targets [64].	Increases memory usage and may introduce redundant features.
Technical Capability	Symmetric encoder–decoder structure [1,65]	Classical U-Net design with down-sampling encoder and upsampling decoder in mirror form.	Balances global context and local detail and is effective for segmentation tasks.	Less flexible for complex tasks.
Technical Capability	High adaptability	Applicable to multiple modalities (CT, MRI, Ultrasound) [66,67].	Strong cross-modality performance with proper architectures.	Domain shift issues reduce generalization across hospitals [68].
Technical Capability	Transfer learning and pretraining [6]	Uses pretrained weights for initialization.	Speeds up convergence, reduces annotation cost, enables performance with limited data [51].	Pretrained models may not always transfer well to medical imaging tasks [69].
Technical Capability	Multi-scale feature learning capability [70,71]	Models extract features at different resolutions or receptive fields.	Improves detection of both small lesions and large organs and balances local details with global context.	Increases model complexity and computation and may require careful architectural design.
Technical Capability	Multimodal fusion capability [72]	Combines complementary information from multiple imaging modalities [72].	Improves lesion recognition and diagnostic accuracy.	Sensitive to noise and fusion can be degraded by poor-quality scans.
Practical Challenge	Interpretability	Deep networks often seen as “black-box” models.	Can provide accurate predictions without handcrafted rules.	Lack of interpretability reduces clinician trust and complicates validation [68].
Practical Challenge	Data Dependency	Requires large annotated datasets for training.	With sufficient annotated data, models achieve high accuracy and robustness.	Annotation is costly, time consuming, and requires clinical expertise.
Practical Challenge	Domain Shift and Generalization	Medical images vary across scanners, hospitals, and populations.	Models trained with domain adaptation can improve cross-center performance.	Models often perform poorly in new domains without adaptation.
Practical Challenge	Computational Burden	Training and inference demand high-performance hardware.	With adequate resources, models achieve state-of-the-art results.	High computational and financial costs limit use in resource-constrained hospitals.
Practical Challenge	Clinical Integration [73]	Adapting AI models into real world clinical workflows.	Can reduce clinician workload and improve diagnostic consistency.	Regulatory, ethical, and usability issues slow down clinical adoption.

**Table 4:** Performance Summary and Application Scenarios of U-Net Variants.

Variant Category	Representative Models	Core Innovation	Ideal Application Scenarios	Performance Advantages	Considerations
Enhanced Skip Connections	U-Net++ [3]	Nested, Dense Skip Pathways [63]	Brain Tumor segmentation [80], Multi-organ Edge Segmentation in Abdominal [79], Skin Lesion Segmentation [81], Breast Tumor Segmentation [82]	Improved multi-scale feature fusion and boundary recovery.	Moderate increase in parameters.
Architectural Deepening	DenseU-Net [19], ResUNet [61]	Dense/Residual blocks [83]	Liver Segmentation [20,84], Brain Tumor Segmentation [85], Lung Segmentation of Preschool Children [86]	Improved gradient propagation and feature reuse; reported performance remains task- and dataset-dependent.	Higher computational cost.
Attention Mechanism	Attention U-Net [2]	Spatial attention gates	Skin Lesion Segmentation [87], Brain Tumor segmentation [71], Retinal Vessels Segmentation [88]	Dynamically suppress irrelevant background areas and enhance target features.	Additional gating operations and task-dependent attention quality.
Deformable Convolution [48,89]	Deformable U-Net [4]	Adaptive sampling grid	Retinal vessels [4]	Improved geometric adaptability for thin or irregular structures.	Specialized design.
Transformer Architecture	TransUNet [5], Swin-Unet [6]	Global self-attention	Ultrasound thyroid nodule segmentation [90]; multi-organ CT segmentation [5,6]	Improved long-range contextual modeling.	High computational cost, requires more data.

**Table 5:** Comparison of U-Net Variants and Their Features.

U-Net Variants	Encoder–Decoder Structure	Skip Connection	Attention Mechanism	Deformable Convolution	Transformer Architecture
U-Net [1]	✓	✓			
Deformable U-Net [4]	✓	✓		✓	
Attention U-Net [2,71,87]	✓	✓	✓		
U-Net++ [3,79,80,81,82]	✓	✓			
Swin-Unet [6]	✓	✓			✓
TransUNet [5,90]	✓	✓	✓		✓
**U-Net [1]:** Prototype architecture. Effectively fuses low-level details and high-level semantic information through symmetric encoder–decoder structure [106] and skip connections [49], solving the localization-semantics contradiction in medical image segmentation. Foundation for all subsequent variants.			**DUNet [4]:** Enhances spatial adaptability. Introduces deformable convolution [48,89] in the encoder, enabling the network to adaptively adjust the receptive field, better model geometric deformations and varying-sized targets, and improve feature extraction for irregular structures.		
**Attention U-Net [2]:** Introduces attention gating [50]. Integrates an attention mechanism (Spatial/Channel Attention Gate) in the skip connections, allowing the decoder to dynamically and selectively focus on regions relevant to the target in the encoder’s feature maps while suppressing irrelevant background noise, significantly improving model accuracy and interpretability.			**U-Net++ [3]:** Optimizes feature fusion. Connects encoders and decoders of different depths through dense nested skip pathways, shortening the semantic gap between feature maps, alleviating potential feature incompatibility issues in original skip connections, and obtaining more powerful multi-scale representation capabilities.		
**Swin-Unet [6]:** Exploration of pure Transformer architecture [76]. Uses a hierarchical encoder–decoder based on Swin Transformer blocks [6]. Its shifted window self-attention mechanism efficiently establishes global dependencies and captures long-range contextual information. A representative work combining U-Net with Vision Transformer.			**TransUNet [5]:** Exemplar of Hybrid architecture. Combines the local feature extraction advantages of CNN with the global context modeling capabilities of Transformer. The encoder uses ViT to process serialized image patches, while the decoder fuses CNN features with Transformer features, achieving breakthrough performance in medical segmentation.		

*Note:* ✓ indicates that the corresponding architectural feature is present.

**Table 6:** Literature-Reported Quantitative Comparisons of Representative U-Net Variants Under Defined Experimental Settings.

Group	Dataset/Modality	Segmentation Task	Model	Experimental Setting	Metric	Reported Result
A	Data Science Bowl 2018/Microscopy	Cell nuclei segmentation	U-Net [1]	Same original study	IoU (%)	90.77
A	Data Science Bowl 2018/Microscopy	Cell nuclei segmentation	U-Net++ w/ DS [3]	Same original study	IoU (%)	92.52
A	ASU-Mayo/RGB colonoscopy	Colon polyp segmentation	U-Net [1]	Same original study	IoU (%)	30.08
A	ASU-Mayo/RGB colonoscopy	Colon polyp segmentation	U-Net++ w/ DS [3]	Same original study	IoU (%)	32.12
A	LiTS/CT	Liver segmentation	U-Net [1]	Same original study	IoU (%)	76.62
A	LiTS/CT	Liver segmentation	U-Net++ w/DS [3]	Same original study	IoU (%)	82.90
B	CHASE_DB1/ Fundus	Retinal vessel segmentation	U-Net [1]	Reimplemented baseline; fixed split	F1/AUC (%)	77.83/97.72
B	CHASE_DB1/ Fundus	Retinal vessel segmentation	DUNet [4]	Reimplemented baseline; fixed split	F1/AUC (%)	80.37/98.12
B	CHASE_DB1/ Fundus	Retinal vessel segmentation	U-Net++ [3]	Reimplemented baseline; fixed split	F1/AUC (%)	83.25/97.98
B	CHASE_DB1/ Fundus	Retinal vessel segmentation	Attention U-Net [2]	Reimplemented baseline; fixed split	F1/AUC (%)	83.84/97.98
C	Synapse/CT	Multi-organ segmentation	U-Net [1]	Synapse benchmark	DSC (%)/HD	76.85/39.70
C	Synapse/CT	Multi-organ segmentation	Attention U-Net [2]	Synapse benchmark	DSC (%)/HD	77.77/36.02
C	Synapse/CT	Multi-organ segmentation	TransUNet [5]	Synapse benchmark	DSC (%)/HD	77.48/31.69
C	Synapse/CT	Multi-organ segmentation	Swin-Unet [6]	Synapse benchmark	DSC (%)/HD	79.13/21.55

*Note:* All quantitative values were extracted from the cited published studies rather than generated through new experiments in this review. Group A reports U-Net and U-Net++ results obtained within the same original study and experimental framework [3]. Group B reports baseline models reimplemented under a fixed data-splitting protocol on CHASE_DB1 for fair comparison [129]. Group C summarizes results reported on the same Synapse multi-organ CT benchmark [6]; U-Net and Attention U-Net were reimplemented in that study, whereas the TransUNet result was taken as a previously reported benchmark result. Therefore, numerical comparisons should be made primarily within the same comparison group and dataset, and results across different groups should not be interpreted as a global ranking because of differences in dataset characteristics, preprocessing, data partitioning, implementation details, and training protocols.

**Table 7:** Modality-Specific Imaging Challenges and Architectural Adaptation of Representative U-Net Variants.

Imaging Modality	Modality-Specific Characteristics	Segmentation Consequences	Representative U-Net Modifications	Why the Modification Helps	Remaining Limitations
MRI	Non-standardized image intensity, bias-field inhomogeneity, multi-sequence heterogeneity, and substantial soft-tissue appearance variation.	The same tissue may exhibit spatially inconsistent signal intensities, while lesions such as brain tumors may have weak boundaries and overlap in appearance with surrounding edema.	Attention U-Net [2,71]; U-Net++ [3,80]	Attention gates suppress irrelevant feature responses and emphasize task-relevant regions, while nested multi-scale feature fusion can improve the preservation of weak boundaries and contextual information.	Neither attention nor dense skip connections directly correct MRI bias fields. Bias-field correction, intensity normalization, and cross-sequence harmonization remain necessary.
CT	Relatively standardized Hounsfield-unit information but limited soft-tissue contrast, image noise, partial-volume effects, and acquisition or reconstruction variability.	Small lesions or tumors with attenuation values similar to surrounding tissue may exhibit poorly defined boundaries and are easily confused with adjacent structures.	U-Net++ [3]; TransUNet [5]	Multi-scale skip fusion preserves local boundary information, whereas Transformer-based global-context modeling incorporates anatomical relationships among lesions and surrounding organs.	Architectural improvements do not eliminate acquisition noise or differences in intensity windowing. Consistent HU windowing, normalization, and preprocessing remain important.
Ultrasound	Speckle-like noise, acoustic shadows and artifacts, low signal-to-noise ratio, blurred or discontinuous boundaries, and substantial device- and operator-dependent variability [126].	Local intensities and edges are unstable, making lesion boundaries difficult to distinguish and increasing sensitivity to background interference.	Attention U-Net [2]; U-Net++ [82]; Deformable U-Net [4]	Attention can suppress irrelevant background responses; multi-scale fusion helps recover weak boundaries at different scales; deformable convolution can adapt to irregular target geometry.	These mechanisms cannot directly remove acquisition-dependent speckle or acoustic artifacts. Noise suppression, augmentation, and device-aware normalization are still required.
Fundus Imaging	Thin and branching vascular structures, low vessel-to-background contrast, illumination variation, and substantial differences in vessel scale.	Small vessels and terminal branches may be fragmented or missed, particularly in low-contrast regions.	Deformable U-Net [4]; Attention U-Net [88]	Deformable convolution adapts sampling locations to curved and branching structures, while attention mechanisms help emphasize vessel-related features and suppress background interference.	Extremely small vessels remain difficult to recover, and differences in cameras, illumination, and patient populations can reduce cross-dataset generalization.
Dermoscopy	Variations in illumination and skin color, hair occlusion, heterogeneous lesion texture, and irregular or low-contrast lesion boundaries.	Lesions may be confused with surrounding normal skin, while artifacts and color similarity can distort boundary localization.	Attention U-Net [87]; U-Net++ [81]	Attention emphasizes lesion-related regions and reduces irrelevant background responses, while multi-scale feature fusion improves boundary representation across lesions of different sizes.	Domain shift caused by skin-tone diversity, acquisition conditions, and imaging artifacts remains a major challenge and cannot be resolved by architectural modification alone.
Microscopy	High cell density, touching or overlapping objects, blurred boundaries, noise, and considerable variation in cell size and morphology.	Adjacent cells may be merged during segmentation, while weak boundaries and dense structures make accurate instance delineation difficult.	DenseU-Net [19]; U-Net++ [3]	Dense feature reuse and multi-scale skip fusion preserve fine-grained spatial features and integrate information across different receptive-field scales.	Dense connectivity increases activation-memory requirements, while highly overlapping cells may still require boundary-aware objectives or additional instance-separation strategies.

*Note:* The architectural modifications listed in this table address segmentation-level consequences associated with different imaging modalities. They should not be interpreted as direct solutions to acquisition-specific artifacts. Modality-specific preprocessing, normalization, harmonization, and appropriate data augmentation remain important components of practical segmentation pipelines.

**Table 8:** Future Challenges, Potential Research Directions, and Typical Application Scenarios for U-Net Variants in Two-Dimensional Medical Image Segmentation.

Model	Key Strengths	Limitations	Future Directions	Typical Application Scenarios
U-Net [1]	Simple, efficient, widely used	Limited global context; sensitive to class imbalance	Add global context modules [134], self-/ semi-supervised pretraining	General medical image segmentation
U-Net++ [3]	Dense skip connections [49] better feature reuse	High memory/computation; complex to deploy	Pruning, model compression, efficient architecture	Accurate clinical segmentation
Attention U-Net [2]	Attention gates [50] improve feature selection	Extra computation; unstable on noisy/small datasets	Lightweight attention; hybrid with Transformers	Skin Lesion segmentation [87], Brain Tumor segmentation [71]
Deformable U-Net [4]	Handles geometric variations deformable convolution [48,89]	High GPU demand; unstable offset learning	Efficient deformable modules; hybrid with Transformers [135]	Organ motion tracking [136], variable anatomies
TransUNet [5]	Combines CNN and Transformer representations for global-context modeling [137]	Very resource-intensive; prone to overfitting	Lightweight hybrid; self-supervised pretraining; efficient inference	Multi-organ, large datasets, high resolution
Swin-Unet [6]	Global-local modeling via hierarchical Transformers [138]	Needs very large data; limited window context	Adaptive windows; transfer/few-shot learning; multi-modal integration	Multi-modal or high-resolution segmentation
DenseU-Net [19]	Dense connections improve feature reuse	High memory/computation; possible redundancy	Model compression; efficient dense modules with attention/Transformers [139]	Vessel/boundary-preserving segmentation
ResUNet [61]	Residuals improve training stability	Large model; may propagate irrelevant features	Lightweight residual blocks; pruning and model compression	General segmentation with limited data

## Data Availability

No new data were created or analyzed in this study. Data sharing is not applicable to this article.

## Abbreviations

The following abbreviations are used in this manuscript:

AI	Artificial intelligence
AUC	Area under the curve
CNN	Convolutional neural network
CT	Computed tomography
DSC	Dice similarity coefficient
FCN	Fully convolutional network
MRI	Magnetic resonance imaging
U-Net	U-shaped network
